# Renewable energy targets and unintended storage cycling: Implications for energy modeling

**DOI:** 10.1016/j.isci.2022.104002

**Published:** 2022-03-04

**Authors:** Martin Kittel, Wolf-Peter Schill

**Affiliations:** 1 DIW Berlin, Department of Energy, Transportation, Environment, Mohrenstraße 58, 10117 Berlin, Germany

**Keywords:** Energy modeling, Energy storage, Energy policy, Energy systems, Energy resources, Energy sustainability

## Abstract

To decarbonize the economy, many governments have set targets for the use of renewable energy sources. These are often formulated as relative shares of electricity demand or supply. Implementing respective constraints in energy models is a surprisingly delicate issue. They may cause a modeling artifact of excessive electricity storage use. We introduce this phenomenon as “unintended storage cycling”, which can be detected in case of simultaneous storage charging and discharging. In this paper, we provide an analytical representation of different approaches for implementing minimum renewable share constraints in energy models, and show how these may lead to unintended storage cycling. Using a parsimonious optimization model, we quantify related distortions of optimal dispatch and investment decisions as well as market prices, and identify important drivers of the phenomenon. Finally, we provide recommendations on how to avoid the distorting effects of unintended storage cycling in energy modeling.

## Highlights

• Renewable energy constraints may cause excessive storage use in energy models
• We investigate how this artifact is related to different constraint implementations
• We quantify distortions of model outcomes with a parsimonious optimization model
• We discuss how to avoid unintended storage cycling in energy models

## Graphical abstract

**Figure:**
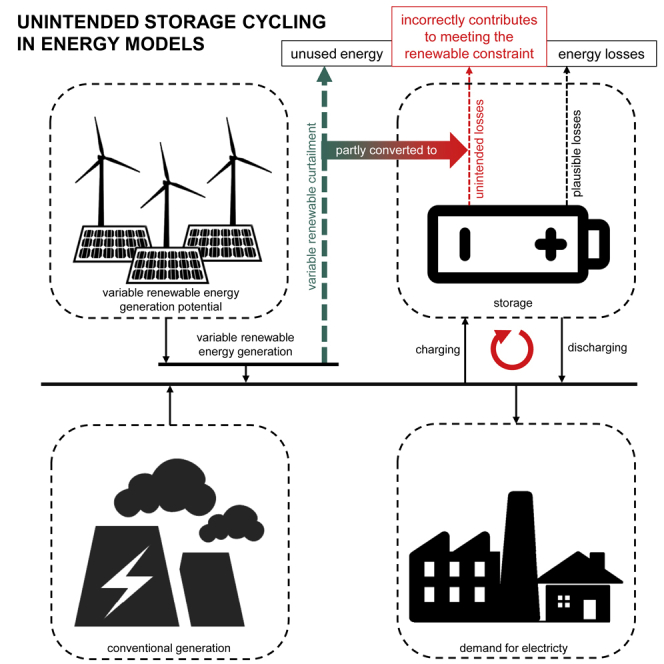

## Introduction

Replacing conventional electricity generation with renewable energy sources is a prime option for mitigating greenhouse gas emissions in the power sector. Technological progress, economies of scale, and support measures have led to substantially growing shares of renewables in power sectors around the world. Firm renewable capacity potentials, i.e., for hydroelectric and biomass power, are limited in many regions. Variable renewable energy (VRE), such as wind and solar photovoltaics (PV), will thus play a major role in the further transition. These technologies have variable generation characteristics, as their temporal availability depends on weather conditions, such as wind speeds and cloud cover (Joskow, 2011). Investment costs for VRE have tremendously decreased in recent years (IEA, 2020). Owing to an insufficient internalization of greenhouse gas emissions (Ricke et al., 2018) and low marginal generation costs of existing thermal power plants, policy support continues to be relevant for low-cost renewable deployment above penetration levels that would emerge without policy intervention (Polzin et al., 2019).

Many governments on state, national, and even intergovernmental levels have adopted a minimum renewable energy share by a target date to promote the deployment of renewable energy (Özdemir et al., 2020). Essentially, this share describes the ratio of energy from renewable sources to the total energy supplied or consumed in a specific economic sector, or in the entire energy system. Such targets and related policy measures can facilitate investment in VRE technologies, and usually rise over time.

For instance, Germany has a long tradition of setting renewable energy targets for its power sector, in particular via the Renewable Energy Sources Act (EEG). The 2021 version of the EEG includes a renewable share of 65% in gross electricity consumption by 2030 (Bundesregierung, 2020). France and Spain aim for a share of 40% and 74% of total generation by 2030, respectively, while Sweden envisages a fully renewable power sector by 2040 (REN21, 2021). In the United States, renewable portfolio standards require a certain share of a utility’s total generation to come from renewable energy sources. In 2020, 30 states had adopted mandatory standards, while in eight states voluntary standards are in place (Zhou and Solomon, 2020). For instance, California aims at a 60% (100%) renewable share by 2030 (2045) (California PUC, 2021). China targets a renewable share of 35% in its total electricity generation by 2030 (REN21, 2020). Japan aims at 22%–24% for the same target year. Overall, 137 countries had renewable energy targets in place for the power sector in 2020 (REN21, 2021). While some of these countries have set only absolute renewable power capacity targets (e.g., Switzerland or New Zealand), most make use of some sort of minimum renewable energy target.

**Figure 1:**
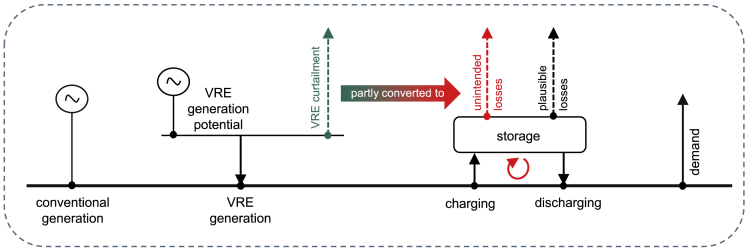
Stylized illustration of unintended storage cycling Instead of curtailing (dashed green) VRE surplus, the optimization may lead to unintended storage cycling (solid red), i.e., to additional storage conversion losses (dashed red), which remove excess renewable electricity from the system.

Capacity expansion models of the power sector or the entire energy system are frequently used to explore future scenarios with high shares of renewables and determine least-cost solutions (Dagoumas and Koltsaklis, 2019). To be policy-relevant, such models are often constrained by renewable energy targets. In this case, an unexpected modeling artifact related to VRE generation may occur. Figure 1 illustrates the general mechanism. At high shares of VRE, situations arise in which the VRE generation potential temporarily exceeds the demand for electricity. In these situations, curtailing the renewable surplus would be an obvious option (dashed green arrow). However, certain minimal renewable share constraint formulations may lead to a situation where renewable curtailment is replaced by storage conversion losses (red dashed error), facilitated by additional, and often simultaneous, storage charging and discharging. Such *unintended storage cycling* drives up generation from VRE compared to the first-mentioned option of curtailing renewable surplus generation, and may thus facilitate achieving renewable energy constraints at lower costs. Note that this mechanism may arise in energy models, but does not relate to real-world storage applications.

The phenomenon of unintended storage cycling has, to the best of our knowledge, not been described or discussed in the energy modeling literature (Bistline et al., 2020). There is reason to believe that this artifact is undetected or ignored, yet prevailing in the power sector and energy system modeling domain. Our paper aims at closing this gap. To this end, we first provide a systematic representation of different approaches for implementing minimum renewable share constraints in energy models. Using a parsimonious optimization model of a power sector (see section on the formal definition of a parsimonious dispatch and investment model), we analytically derive some intuition as to how these constraints may lead to unintended storage cycling and examine their impact on equilibrium conditions of storage operators. For a stylized model parameterization (see the section on the numerical implementation), we numerically analyze how this phenomenon may distort model outcomes. We quantify the effects on dispatch and investment decisions of both storage and other technologies, and on market prices. We also explore important drivers of unintended storage cycling. Finally, we discuss different options for avoiding the distorting effects of unintended storage cycling in energy modeling and provide recommendations for energy modelers. Additional material is available in the supplemental information.

In energy modeling applications, constraints requiring a certain renewable share may be manifold. A review by Gillich et al. (2019) shows that some applications use constraints for installed renewable generation capacity (e.g., in GW). Other model applications use constraints on the share of renewable energy (e.g., in TWh). Table 1 shows a selection of well-established European models and model applications that employ renewable capacity and energy targets. Additionally, two commonly implemented climate policy instruments are listed, which also influence the share of renewable energy sources in model outcomes: an annual greenhouse gas emission budget and an explicit CO_2_ price. The information is based on a review of research articles, model documentations as well as other references such as dissertations, and, wherever possible, on personal validation by model developers or affiliated modelers.

**Table 1:** Model features that affect the shares of renewable energy sources in selected European energy models and model applications

Model	Renewable share in demand	Conventional share in demand	Renewable share in generation	Conventional share in generation	Renewable capacity target	CO_2_ budget	CO_2_ price	Documentation
AnyMOD						x^a^	x^a^	Göke (2021)
Backbone∗	x^a^	x^a^			x^a^	x^a^	Helistö et al. (2020, 2021); Lindroos et al. (2021);Ikäheimo et al. (2022); Rasku et al. (2020)	Helistö et al. (2019)
Calliope∗	Tröndle (2020), x^b^	x^a^	x^a^	x^a^	Pfenninger and Keirstead (2015); Pfenninger (2017)	Pickering and Choudhary (2021)	Pickering and Choudhary (2021)	Pfenninger and Pickering (2018)
DIETER∗		Zerrahn and Schill (2017); Schill et al. (2017b); Schill and Zerrahn (2018)	Schill and Zerrahn (2020); Stöckl et al. (2021)			x^b^	x^b^	Zerrahn and Schill (2017); Gaete-Morales et al. (2021)
DIMENSION∗	x^c^	x^c^	x^c^	x^c^	Bründlinger et al. (2018)	Helgeson and Peter (2020)	x^a^	Richter (2011); Helgeson and Peter (2020)
dynELMOD∗					Kittel et al. (2020)	Gerbaulet et al. (2019)		Gerbaulet and Lorenz (2017)
EMMA∗	x^b^				Hirth (2013); Ruhnau et al. (2020); Ruhnau (2021); Ruhnau et al. (2021)	x^a^	Hirth (2013); Ruhnau et al. (2020); Ruhnau (2021); Ruhnau et al. (2021)	Hirth (2013)
ELTRAMOD					Anke and Möst (2021)	Anke et al. (2020); Hobbie et al. (2019)	Eising et al. (2020)	Ladwig (2018)
EU-REGEN∗			x^a^			Mier and Weissbart (2020); Weissbart (2020); Azarova and Mier (2021)	Mier and Azarova (2021); Siala et al. (2020)	Weissbart and Blanford (2019)
E2M2∗	Fleischer (2019)		Torralba-Díaz et al. (2020)		Schick et al. (2020)	Gillich et al. (2019); Fleischer (2019); Guthoff et al. (2021); Torralba-Díaz et al. (2020)	Torralba-Díaz et al. (2020); Schick et al. (2020)	Fleischer (2019)
ENTIGRIS∗	Senkpiel et al. (2019)	Senkpiel et al. (2019)					Senkpiel et al. (2019)	n/a
GENESYS						x^c^	x^c^	Bussar et al. (2014); Bussar (2019)
GENeSYS-MOD∗			Loeffler et al. (2017)			Hainsch et al. (2021)	Burandt et al. (2018)	Burandt et al. (2018)
ISAaR						x^a^	x^c^	Böing and Regett (2019)
LIMES-EU∗	Pietzcker et al. (2021)		x^a^			Pietzcker et al. (2021)	Osorio et al. (2020b)	Osorio et al. (2020a); Knopf et al. (2015)
LUT Model∗	x^b^	Bogdanov et al. (2021)	SolarPower Europe and LUT University (2020)	SolarPower Europe and LUT University (2020)	Renewable Energy Institute, Agora Energiewende, LUT University (2021)	x^a^	Satymov et al. (2021)	Bogdanov et al. (2021)
oemof	x^c^	x^c^			Hilpert et al. (2020)	x^c^	x^c^	Hilpert et al. (2020)
PLEXOS∗	x^c^	x^c^	x^c^	x^c^	x^c^	x^c^	x^c^	Deane et al. (2015)
PyPSA∗	Brown and Reichenberg (2021)		x^c^		x^c^	Brown and Reichenberg (2021); Parzen et al. (2021)	Neumann and Brown (2021)	Brown et al. (2018a)
REMix∗	Xiao et al. (2020)		Gils et al. (2017); Scholz et al. (2017)			Moser et al. (2020)	Gils et al. (2019)	Gils et al. (2017); Scholz et al. (2017)
SpineOpt.jl	x^a^	x^a^			x^a^	x^a^	x^a^	Ihlemann et al., 2021

*Notes:* ∗ Personal communication confirms that the artifact has been observed in this model.

a Possible, but not used or documented in model applications so far.

b Used in model applications, but not published yet.

c Applicable according to personal communication, but insufficient information for linking specific model applications to this particular constraint available.

Renewable energy constraints in power sector and energy system models may come in various forms. Energy provision from renewable (conventional) energy sources has to achieve (must not exceed) a certain share in total demand or generation within a particular sector, or in the entire energy system. Additionally, storage losses may be covered to a different extent in such constraints. Based on this survey, we systematize possible ways of implementing renewable energy targets, and examine their impact on modeling results.

The artifact of unintended storage cycling may occur in all cost-optimizing power sector and energy system models that include binding renewable targets. Personal communication further confirms that the phenomenon has already been observed in various European energy models, which are marked with an asterisk (∗) in Table 1. For entries without an asterisk, it was not possible to clarify whether the artifact has been observed so far. Albeit the artifact has been observed, we do not claim that it prevails in the listed applications.

Not all of the listed models consider storage losses in the formulation of the renewable target, and only a minority does so to the full extent. We discuss how this impacts the results of linear optimization models. As we show in this paper, an incomplete consideration of storage losses in the renewable energy constraint causes unintended storage cycling.

### An illustration of different types of unintended storage cycling

Unintended storage cycling can be observed in case of simultaneous charging and discharging of storage. This involves energy that is cycled through the storage within the same period, which we refer to as same-period cycling (SPC). Further, there is also an inter-temporal instance of the artifact: Across-period cycling (APC). It refers to a situation in which unintendedly discharged energy has been charged prior to the period of unintended discharge. Both SPC and APC are excessive and constitute unintended storage use.

**Figure 2:**
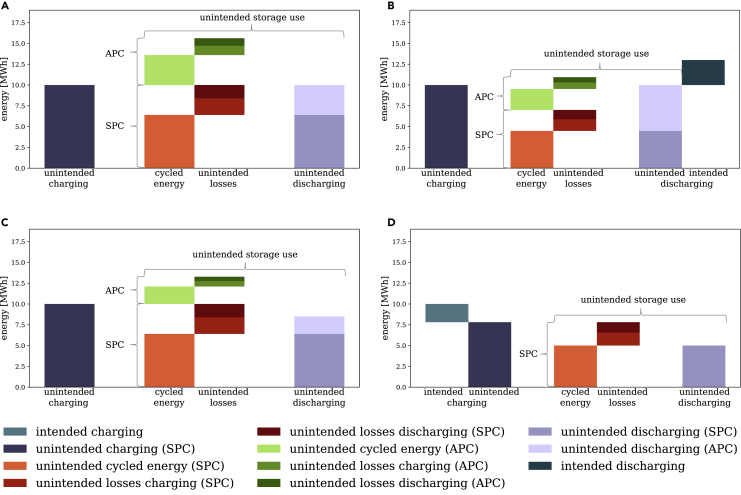
Four types of unintended storage cycling (A) Type 1: Storage charging equals discharging. (B) Type 2: Storage discharging exceeds charging. (C) Type 3: Storage charging exceeds discharging; discharging exceeds same-period unintendedly cycled energy. (D) Type 4: Storage charging exceeds discharging; but not the full cycling potential is exploited.

There are four conceivable types of unintended storage cycling. They differ in terms of the ratio of storage charging to discharging within the same period. Further, they vary in terms of the temporality of unintendedly cycled energy, which may be cycled either within the period (SPC only), or within the same and across periods (SPC and APC). Figure 2 illustrates these types with stylized numbers. For the sake of illustration, they are based on an assumed low efficiency for charging and discharging of 80%, respectively.

The first type occurs in a situation in which storage charging and discharging within the same period are equal (Figure 2A). Owing to conversion losses, out of 10 MWh charged energy (left bar) only 6.4 MWh can be discharged (dark area, right bar). This energy amount represents the unintendedly cycled energy within the same period (orange area, middle bar). Corresponding losses when charging and discharging amount to 2.0 and 1.6 MWh (light and dark red areas, middle bar), respectively. We define the total energy required for cycling these 10 MWh within the same hour as SPC. It comprises the unintendedly cycled energy (6.4 MWh) plus conversion losses (3.6 MWh in total), totaling 10 MWh. To achieve a total discharge of 10 MWh in this period, it takes another 3.6 MWh (lighter area, right bar) of energy that already had to be stored during a previous period. This additional unintendedly cycled energy of 3.6 MWh (light green, middle bar) is cycled across periods. Again, it entails storage conversion losses when charging (1.125 MWh, medium green, middle bar) and discharging (0.9 MWh, dark green, middle bar). We define the energy for unintended cycling across periods (3.6 MWh plus 2.025 MWh losses), totaling 5.625 MWh, as APC. The total energy required for equal storage charging and discharging of 10 MWh within the same period is 25.625 MWh. It contains the total charging, which is the sum of SPC and APC (15.625 MWh), plus the total unintended discharging (10 MWh). The net effect of such storage use on the system’s energy balance in the respective hour is zero. Thus, the entire 25.625 MWh are considered as unintended storage use. We then define unintended storage losses as the energy removed from the system through SPC and APC losses. Essentially, unintended storage cycling transforms VRE surplus into unintended storage losses and circumvents renewable curtailment. In our running example, the unintended storage loss is 5.625 MWh.

The second type of unintended storage cycling occurs when storage discharge exceeds the same-period charging, i.e., effective discharging (Figure 2B). Both SPC and APC occur in this case, similar to the first type. However, the difference between the same-period charging and discharging (dark area, right bar) exerts a net effect on the system’s energy balance in this period. This is intended storage use that helps to serve demand, and needs to be distinguished from storage use solely arising to generate additional losses to remove excess electricity from the system.

The third and fourth types of unintended storage cycling occur when more energy is charged than discharged within the same period. In the third type, total discharged energy consists of both SPC and APC, with less use of APC compared to the first type (Figure 2C). In the fourth type, effective charging occurs. There is no APC, and the unintended cycling potential is not necessarily fully exhausted (Figure 2D). In the depicted case, the SPC is smaller than the charged energy, leaving room for some level of intended charging.

## Results

### An analytical approach to unintended storage cycling

#### Alternative renewable energy constraint formulations

Renewable energy constraints in energy models may be generalized as follows:

(Equation 1) $$base\;element⋛share^{ref}\times reference\;element\pm share^{loss}\times losses$$

Varying the base and reference element, four basic constraint formulations are possible. For the sake of argument, we denote them *constraint families*: (1) a minimum renewable share in total demand, (2) a minimum renewable share in total generation, and, conversely, (3) a maximum conventional share in total demand, and (4) a maximum conventional share in total generation. Note that total generation includes storage losses, while total demand does not. Generation from renewables in constraint families (1) and (2) may include both firm and variable renewable energy sources. Formulations (3) and (4) indirectly enforce minimum renewable shares, as the limited generation from conventional technologies has to be complemented by renewables.

Reflecting different practices in the modeling literature, we investigate three variations for each constraint family as to what degree renewable generators cover storage losses. We denote this characteristic *Storage Loss Coverage by Renewables (SLCR)*. Let *φ* be the targeted renewable share, the SLCR then may take the following values: (a) storage losses are completely supplied from conventional energy sources (*zero SLCR*); (b) storage losses are shared between renewable and conventional energy sources according to the targeted renewable share *φ* and (1−φ), respectively (*proportionate SLCR*); or (c) storage losses are completely covered by renewables (*complete SLCR*).

Given current economics, power sector costs generally increase with VRE penetration, as the need for providing temporal flexibility rises. Note that we are interested in settings with high VRE penetration where the renewable energy constraint is binding, i.e., with a penetration beyond the optimal share of an unconstrained model without an explicit renewable energy constraint. Model specifications with zero SLCR are the least restrictive ones, as these require the lowest use of VRE. In contrast, models with complete SLCR are most restrictive, as these require storage losses to be completely covered by VRE. Model specifications with proportionate SLCR may appear most intuitive and appropriately restrictive. Yet, we show that incomplete SLCR levels lead to unintended storage cycling.

In total, the four constraint families and three SLCR levels yield twelve different *model specifications* (Table 2). The nomenclature is introduced in the section on the formal definition of a parsimonious dispatch and investment model.

**Table 2:** Investigated constraint formulations differentiated by constraint family and SLCR level

Model specification	Base element	Sign	Share^*ref*^ & reference element	±	Share^*loss*^ storage losses	Associated constraint
1a	$\underset{s\in R,t}{\sum }G_{s,t}$	≥	$\varphi \sum_{t}d_{t}$			(10i)
1b	$\underset{s\in R,t}{\sum }G_{s,t}$	≥	$\varphi \sum_{t}d_{t}$	+	$\varphi \underset{r,t}{\sum }\left(G_{r,t}^{in}-G_{r,t}^{out}\right)$	(10i)
1c	$\underset{s\in R,t}{\sum }G_{s,t}$	≥	$\varphi \sum_{t}d_{t}$	+	$\underset{r,t}{\sum }\left(G_{r,t}^{in}-G_{r,t}^{out}\right)$	(10i)
2a	$\underset{s\in R,t}{\sum }G_{s,t}$	≥	$\varphi \underset{s,t}{\sum }G_{s,t}$	−	$\varphi \underset{r,t}{\sum }\left(G_{r,t}^{in}-G_{r,t}^{out}\right)$	(10i)
2b	$\underset{s\in R,t}{\sum }G_{s,t}$	≥	$\varphi \underset{s,t}{\sum }G_{s,t}$			(10i)
2c	$\underset{s\in R,t}{\sum }G_{s,t}$	≥	$\varphi \underset{s,t}{\sum }G_{s,t}$	+	$\left(1-\varphi \right)\underset{r,t}{\sum }\left(G_{r,t}^{in}-G_{r,t}^{out}\right)$	(10i)
3a	$\underset{s\in C,t}{\sum }G_{s,t}$	≤	$\left(1-\varphi \right)\underset{t}{\sum }d_{t}$	+	$\underset{r,t}{\sum }\left(G_{r,t}^{in}-G_{r,t}^{out}\right)$	(10j)
3b	$\underset{s\in C,t}{\sum }G_{s,t}$	≤	$\left(1-\varphi \right)\underset{t}{\sum }d_{t}$	+	$\left(1-\varphi \right)\underset{r,t}{\sum }\left(G_{r,t}^{in}-G_{r,t}^{out}\right)$	(10j)
3c	$\underset{s\in C,t}{\sum }G_{s,t}$	≤	$\left(1-\varphi \right)\underset{t}{\sum }d_{t}$			(10j)
4a	$\underset{s\in C,t}{\sum }G_{s,t}$	≤	$\left(1-\varphi \right)\underset{s,t}{\sum }G_{s,t}$	+	$\varphi \underset{r,t}{\sum }\left(G_{r,t}^{in}-G_{r,t}^{out}\right)$	(10j)
4b	$\underset{s\in C,t}{\sum }G_{s,t}$	≤	$\left(1-\varphi \right)\underset{s,t}{\sum }G_{s,t}$			(10j)
4c	$\underset{s\in C,t}{\sum }G_{s,t}$	≤	$\left(1-\varphi \right)\underset{s,t}{\sum }G_{s,t}$	−	$\left(1-\varphi \right)\underset{r,t}{\sum }\left(G_{r,t}^{in}-G_{r,t}^{out}\right)$	(10j)

These constraint formulations base on Equation (1). The right-hand sides of the inequalities define Θ or Ω of the optimization problem introduced in the section on the formal definition of a parsimonious dispatch and investment model.

#### Intuition for unintended storage cycling

We first provide some intuition for model specification (1a), i.e., a model using a minimum renewable share in demand. Suppose the optimal renewable share of an unconstrained model is below $\varphi \sum_{t}d_{t}$. Introducing constraint (10i), forces the model to increase renewable generation. Further, suppose there are hours with renewable curtailment. There are two options to increase $\underset{s\in R,t}{\sum }G_{s,t}$. Either increase renewable generation capacity, which incurs additional investment costs, or transform renewable curtailment into storage losses, which only incurs variable storage costs. The latter is done by increasing $G_{s\in R,t}$ in curtailment hours, and by subsequently charging and discharging storage in these hours. This leads to additional storage losses from unintended storage cycling, which are not accounted for in the renewable constraint formulation of model specification (1a). Suppose demand is 100 MWh in one of these curtailment hours, and storage charging and discharging capacity is 10 MW with a round-trip efficiency of 80%. Without unintended storage cycling, $G_{s\in R,t}$ in this hour is 100 MWh. Making use of unintended storage cycling, $G_{s\in R,t}$ can increase to 102 MWh, consisting of 10 MWh charged into storage and 92 MWh to satisfy demand. In the same hour, 8 MWh are discharged from storage to serve the load of 100 MWh. In this example, renewable generation increases by 2 MWh compared to a setting without unintended storage cycling. This is done without additional renewable investments, but by transforming 2 MWh of renewable curtailment into storage losses, which allows us to meet constraint (10i), i.e., $\underset{s\in R,t}{\sum }G_{s,t}\geq \varphi \underset{t}{\sum }d_{t}$, at lower costs. Note that the additional 2 MWh of renewable generation contribute to meeting the renewable energy target (Equation 10i), but do not serve electricity demand (Equation 10b), as the energy is lost in the storage conversion process.

Under model specification (1b), a similar reasoning applies. The only difference is that storage losses are partially accounted for in the renewable constraint with $\varphi \underset{r,t}{\sum }\left(G_{r,t}^{in}-G_{r,t}^{out}\right)$. Only the remaining fraction of storage losses, $\left(1-\varphi \right)\underset{r,t}{\sum }\left(G_{r,t}^{in}-G_{r,t}^{out}\right)$, can be used to transform curtailment into storage losses via unintended storage cycling. Suppose the targeted renewable share is φ=80% in the setting described above. Unintended storage cycling still leads to a renewable generation increase of 2 MWh in the exemplary hour, which is transformed into storage losses. Yet, 80% of these losses are accounted for in the renewable constraint. Thus, only 20% of the increase in renewable generation, i.e., 0.4 MWh, helps to relax the renewable constraint. To achieve a similar effect as under model (1a), more unintended storage cycling would be required.

Under model specification (1c), storage losses are completely covered by the renewable energy constraint. Unintended storage cycling would only increase total variable storage costs, without providing any benefit in terms of meeting the renewable constraint. Accordingly, renewable generation can only be increased via additional renewable capacity investments.

A similar reasoning applies to the other renewable share constraints listed in Table 2. For models using constraint family (2), the only difference is that the fraction of storage losses covered by renewables is slightly less intuitive than in the example described above, as storage losses are already included in overall generation $\underset{s,t}{\sum }G_{s,t}$. The effects in constraint families (3) and (4) are similar to the ones in (1) and (2), respectively.

#### Long-term equilibrium conditions for storage

We first consider an unconstrained long-term equilibrium of the model defined in the section on the long-term equilibrium conditions for storage without binding renewable energy targets, i.e., discarding constraints (10j) and (10i). Joskow (2011) stresses that both cost and value of a technology matter in such a setting. The levelized cost of storage (LCOS) of a storage unit *r* is the average cost of each unit energy discharged (Pawel, 2014). It includes investment and variable costs as well as the cost of the electricity used for charging. As it relates to the storage output, the LCOS also consider the value of conversion losses.

(Equation 2) $$LCOS_{r}=\frac{\sum_{\circ }i_{r}^{\circ }C_{r}^{\circ }+\sum_{t,*}o_{r}^{\text{∗}}G_{r,t}^{\text{∗}}+\sum_{t}\lambda_{t}G_{r,t}^{in}}{\sum_{t}G_{r,t}^{out}}$$

When interpreting the LCOS metrics, the temporal heterogeneity of electricity as a good has to be considered. The value of electricity is time-variant due to a number of reasons (Joskow, 2011; Hirth, 2013; Ueckerdt et al., 2013). Both the electricity demand and the output of many generation technologies are fluctuating in nature. Especially, the value of VRE depends on the time when they generate, i.e., their variable availability profile (López-Prol and Schill, 2021), and the location of generation in the geographical context. Consequently, the electricity price fluctuates over time and space, too. Transmission and distribution grid constraints might further affect the locational value of VRE.

The market value (MV) accommodates these variations in electricity value (Joskow, 2011). The MV of a storage technology *r* refers to the average value of each unit of energy supplied to the system. Storage operators generate revenue by dispatching stored energy at market prices $\lambda_{t}$. They exploit arbitrage opportunities, ideally dispatching during periods with high prices, while charging during low-price periods (Brown and Reichenberg, 2021).

(Equation 3) $$MV_{r}=\frac{\sum_{t}\lambda_{t}G_{r,t}^{out}}{\sum_{t}G_{r,t}^{out}}$$

Storage achieves economic efficiency if LCOS equals its marginal economic value, and competitiveness if LCOS is less or equal to its MV. For perfect and complete markets, the marginal economic value and the MV coincide (Ueckerdt et al., 2013). In the long-term equilibrium of an unconstrained optimum, consequently, LCOS equals storage MV, i.e., storage operators generate zero profit as costs perfectly match revenue (zero-profit condition) (Brown and Reichenberg, 2021). Using the Lagrangian function and Karush-Kuhn-Tucker (KKT) conditions (Section SI.3), Section SI.4 proves this optimality condition.

(Equation 4) $$LCOS_{r}=MV_{r}$$

Imposing a binding renewable energy target, i.e., including constraints (10j) and (10i), may alter the zero-profit condition of storage in a long-term equilibrium. For clarity, we define a new metric, the normalized storage conversion losses (NSL) of a storage unit *r*. It indicates storage conversion losses related to each unit of storage output.

(Equation 5) $$NSL_{r}=\frac{\sum_{t}\left(G_{r,t}^{in}-G_{r,t}^{out}\right)}{\sum_{t}G_{r,t}^{out}}$$

Table 3 shows that a binding renewable energy target may affect the composition of storage costs and revenue streams of all investigated model specifications. Exemplary proofs of those conditions can be found in Section SI.5.

**Table 3:** Storage operators' zero-profit conditions

model	Costs		revenue
1a	$LCOS_{r}$	=	$MV_{r}$
1b	$LCOS_{r}+\varphi \mu_{\theta }NSL_{r}$	=	$MV_{r}$
1c	$LCOS_{r}+\mu_{\theta }NSL_{r}$	=	$MV_{r}$
2a, 4a	$LCOS_{r}-\varphi \mu_{\theta }NSL_{r}$	=	$MV_{r}$
2b, 4b	$LCOS_{r}$	=	$MV_{r}$
2c, 4c	$LCOS_{r}+\left(1-\varphi \right)\mu_{\theta }NSL_{r}$	=	$MV_{r}$
3a	$LCOS_{r}-\mu_{\omega }NSL_{r}$	=	$MV_{r}$
3b	$LCOS_{r}-\left(1-\varphi \right)\mu_{\omega }NSL_{r}$	=	$MV_{r}$
3c	$LCOS_{r}$	=	$MV_{r}$

Within each constraint family, the LCOS increase by a certain margin, the more storage losses are covered by renewables. This margin is a multiple of the factor $\mu_{\theta /\omega }NSL_{r}$, which represents the storage loss per unit storage output, valued at the marginal system costs of an additional unit of renewable generation. For a binding renewable target, renewable energy becomes a scarce good with additional inherent value. When renewable energy is lost in storage conversion processes, its inherent value for achieving the renewable constraint is annihilated. Restoring the lost energy incurs additional costs. We thus interpret this factor as storage integration costs that arise in a system with a binding renewable energy constraint. If the constraint is not binding, the dual variable of the minimum renewable constraint $\mu_{\theta /\omega }$ is zero, and the zero-profit condition of storage remains unchanged compared to the unconstrained optimum without renewable policy targets.

Models with complete SLCR include all storage losses arising in the system in the formulation of the renewable energy constraint. The zero-profit condition of storage operators properly accounts for all storage loss costs related to the binding renewable target. The economic trade-off between storage use and renewable curtailment is properly specified such that unintended storage cycling is prevented.

In contrast, models with zero SLCR neglect storage losses in the minimum renewable constraint. They can be completely covered by conventional generators. Consequently, the zero-profit condition of storage operators does not include any storage loss costs related to the binding renewable target. Within the same constraint family, the zero-profit condition is reduced by the factor $\mu_{\theta /\omega }NSL_{r}$ compared to models with complete SLCR. Similarly, models with proportionate SLCR only partially require renewables to cover storage losses. The system costs of storage losses are thus only partially imposed on storage operators in the long-term equilibrium. Compared to models with complete SLCR within the same constraint family, the zero-profit condition is reduced by the factor $\left(1-\varphi \right)\mu_{\theta /\omega }NSL_{r}$, which corresponds to the share of storage loss costs that is not accounted for in the renewable constraint. In other words, models based on zero and proportionate SLCR do not fully account for the underlying LCOS related to the binding renewable target. This causes excessive storage use and too little VRE surplus curtailment in the long-term equilibrium.

### Numerical results for unintended storage cycling

#### Occurrence and effects on total system costs

**Figure 3:**
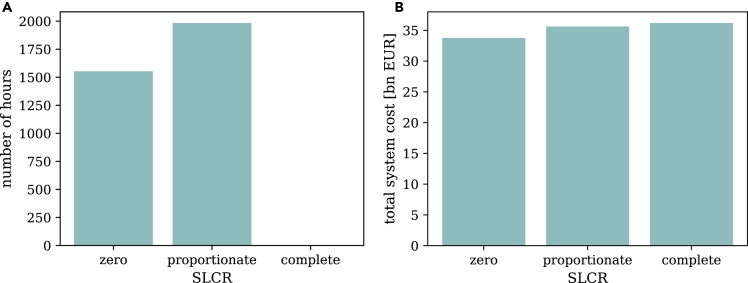
Number of hours in which unintended storage cycling occurs and total system costs per SLCR level at a minimum renewable share of 80% These graphs are similar for all constraint families, with only marginal differences. (A) Number of hours in which unintended storage cycling occurs. (B) Total systems costs.

We test 12 different model specifications with alternative minimum renewable share constraint formulations as detailed in Table 2, using the stylized numerical model introduced in the section on the numerical implementation. We find that unintended storage cycling is a frequently occurring modeling artifact. It arises when the SLCR level of the imposed renewable share constraint is incomplete (i.e., constraints with suffix a and b in Table 2), regardless of the constraint family (1, 2, 3, or 4). This is most pronounced for models with proportionate SLCR. Only models with complete SLCR (constraints c) prevent the model artifact (Figure 3A), i.e., requiring renewable generation to completely cover storage losses averts unintended storage cycling. We further find the fourth type of unintended storage cycling to be most common. Most model outcomes vary across SLCR levels (i.e., constraints with suffix a, b, or c), but coincide across constraint families. For the sake of illustration, we thus present our findings differentiated by SLCR level, unless stated otherwise.

Confirming the intuition laid out in the previous sections, the artifact leads to lower-cost solutions (Figure 3B). This essentially happens as the renewable constraint is partly relaxed in the sense that storage losses also contribute to meeting the renewable target. Compared to the setting with complete SLCR, some renewable curtailment is avoided and transformed into additional VRE generation, and subsequently, into storage losses. However, this energy is not used to serve final demand—which is clearly an unintended effect of the renewable constraint formulation.

#### Effects on optimal dispatch and investment decisions

**Figure 4:**
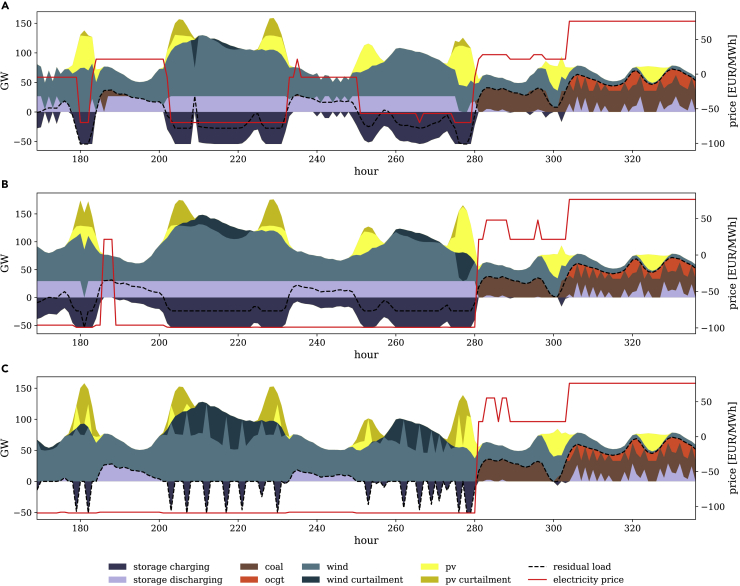
Generation and price profiles for the second week of the target year at a renewable target of 80% in total demand The positive part of the ordinate relates to storage discharging, its negative part to charging. (A) Model specification (1a) with zero SLCR. (B) Model specification (1b) with proportionate SLCR. (C) Model specification (1c) with complete SLCR.

Figure 4 illustrates generation, curtailment, and storage use for an exemplary week for model specifications imposing a minimum renewable share in demand with zero SLCR (1a), proportionate SLCR (1b), and complete SLCR (1c). Respective illustrations for the other three constraint families (2, 3, and 4) are very similar with only marginal deviations. In the models with zero and proportionate SLCR, unintended storage cycling takes place, e.g., between hours 200 and 230, but not in the model with complete SLCR. The artifact’s impact is not limited to the simultaneity of charging and discharging. The hourly storage usage patterns also differ substantially.

Unintended storage cycling also affects the generation profiles of renewable and conventional generators, as well as residual load. The latter is demand for electricity in a given time period, e.g., 1 h, net of VRE generation potential during this period. A positive residual load refers to situations in which demand exceeds the VRE generation potential, and requires the use of dispatchable generation technologies. In contrast, the residual load is negative if the VRE generation potential exceeds demand. Residual load in the exemplary week is lower during periods of renewable curtailment in models with zero or proportionate SLCR compared to models with complete SLCR. This is a consequence of lower curtailment of renewable generation, notably during the first days of the week, which is transformed into additional storage losses related to unintended storage cycling. During the last days of the week, renewable energy is scarce as a result of prevailing weather conditions, and residual load has to be met by dispatchable power plants. Generation from conventional technologies in models with zero or proportionate SLCR slightly exceeds their complete SLCR equivalent. This is caused by lower VRE capacity in the models with incomplete SLCR.

**Figure 5:**
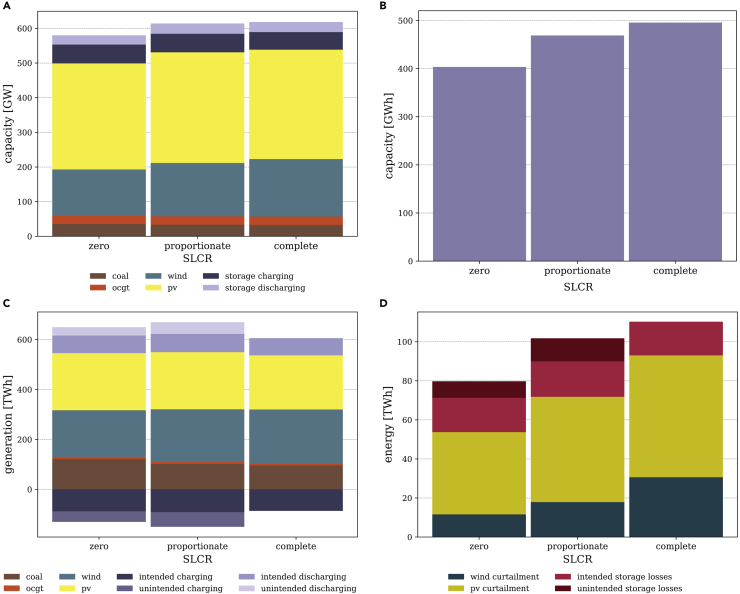
Installed capacity, annual generation, curtailment, and storage losses per technology at a renewable share of 80% (A) Installed generation capacity including storage charging and discharging. (B) Installed storage energy capacity. (C) Generation including storage charging (negative part of ordinate) and discharging. (D) Storage losses and curtailment.

Unintended storage cycling also distorts optimal capacity and annual dispatch decisions. The effects are largely similar in models using the same SLCR level across all constraint families. In models with incomplete SLCR, VRE generation capacity (Figure 5A) and storage energy capacity (Figure 5B) decrease, while the dispatch of conventional generators as well as storage use increase (Figure 5C). Renewable curtailment declines and is partly converted into unintended storage losses (Figure 5D). Note that while investment decisions coincide across all constraint families (Figure S2), constraint family (3) models use a bit less wind power and more PV (Figure S1).

If unintended storage cycling occurs, the increase in total storage charging and discharging is related to unintended storage use (Figure 5C), generating unintended storage losses (Figure 5D). This effect is most pronounced in models with proportionate SLCR, as only the fraction of the additional storage losses helps to achieve the renewable target that is not covered by the renewable energy constraint (compare the section on the intuition for unintended storage cycling). About 96% of the unintended storage use relates to SPC, while APC accounts for the remaining 4%.

Renewable curtailment decreases due to the conversion of renewable curtailment into additional VRE generation, and subsequently, unintended storage losses. This substitution is most effective in models with zero SLCR. Because of the additional renewable generation that is not curtailed, but removed via unintended storage cycling, much less VRE capacity is required in models with incomplete SLCR to meet the renewable energy constraint (Figure 5A). This is most pronounced for wind power, which is the most expensive renewable energy source under the parameterization used here. PV capacity slightly increases, while optimal wind power deployment disproportionately decreases compared to the case with complete SLCR. The same holds true for generation from both technologies (Figure 5C). Renewable curtailment decreases further as a consequence of the capacity effect. Accordingly, renewable surplus energy declines, such that the need for storing excess energy over a longer period of time decreases, which yields a lower optimal storage energy capacity (Figure 5B).

Renewable energy that is removed from the system in the form of unintended storage losses contributes to the renewable energy constraint. However, it does not provide useful energy for serving demand. To meet the system’s energy balance, generation from coal plants thus increases during hours in which, due to the VRE capacity effect, the VRE generation potential decreases compared to a setting without unintended storage cycling. This is most pronounced for models with zero SLCR. Because of the increase in generation from coal plants, carbon dioxide emissions grow by 25% or 6% in models with zero or proportionate SLCR, respectively.

Aside from effects directly related to unintended storage cycling, the developments illustrated in Figure 5 are also driven by a lower ambition level of the renewable energy constraints of models with zero or proportionate SLCR. Because storage losses can, at least partly, be covered by conventional generators here, these models require lower renewable generation than a specification with complete SLCR by definition. Section SI.7 disentangles the impact of both factors. The factor separation raises complementary insights, but also introduces new complexities (Figure S3). For practical model applications, only the combined effects of varying renewable ambition levels and unintended storage cycling are relevant, which are shown in Figure 5.

#### System effects of storage illustrated with residual load duration curves

**Figure 6:**
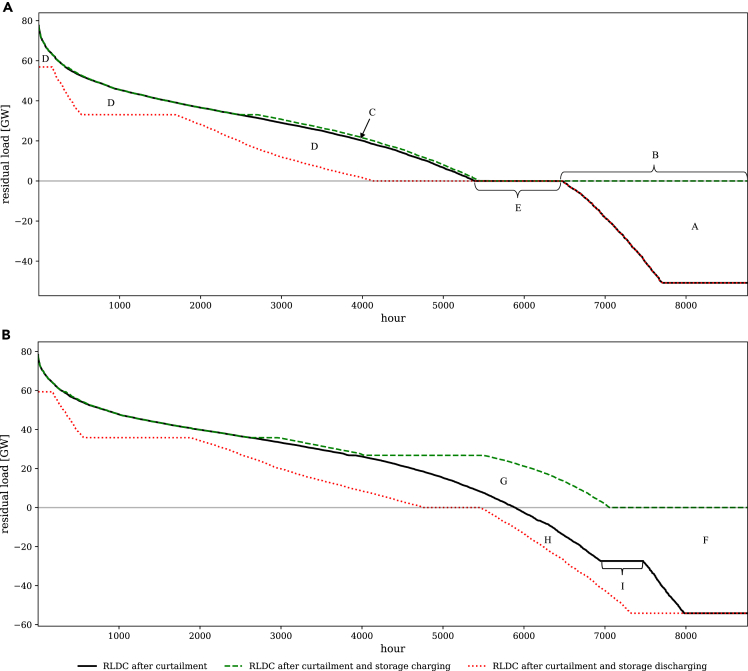
Different configurations of the RLDC at a targeted renewable share of 80% (A) Model specification (1c) with complete SLCR. (B) Model specification (1b) with proportionate SLCR.

**Figure 7:**
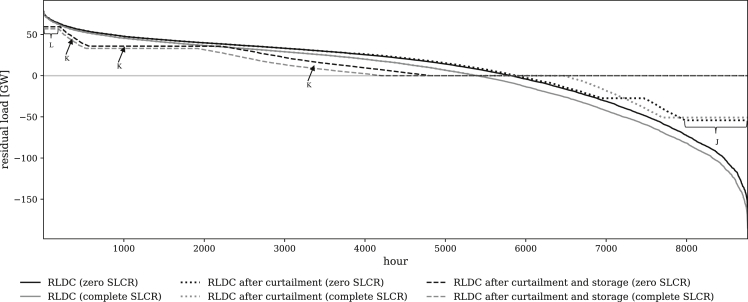
Different configurations of the RLDC for the model specification (1a) with zero SLCR in black and the specification (1c) with complete SLCR in gray at a targeted renewable share of 80%

To illustrate system effects of electricity storage and VRE, a residual load duration curve (RLDC) can be used (Ueckerdt et al., 2015; Zerrahn et al., 2018; Schill, 2020). A standard RLDC sorts all hourly residual load values of a full year in descending order (solid lines in Figure 7). With increasing VRE penetration, the RLDC shifts downward especially on the right-hand side (López-Prol and Schill, 2021). Augmented versions of residual load duration curves can be calculated that also consider optimal curtailment and electricity storage use (Figures 6 and 7).

In a case without unintended storage cycling, the optimum storage takes up a certain amount of renewable surplus generation (area A in Figure 6A, leading to the plateau B) as well as some generation from dispatchable power plants with low variable costs (area C). Storage shifts this energy to periods with positive residual load and VRE shortage (area D), displacing conventional generation. Optimal renewable curtailment yields a number of situations with zero residual load in which storage is not being used (plateau E of the RLDC after curtailment in Figure 6A).

Accordingly, RLDCs illustrate the trade-off between optimal storage sizing and VRE curtailment. As storage investments incur costs, it is not optimal to fully take up renewable surplus generation, but to also allow some VRE curtailment. In general, this lowers the need for both storage charging and energy capacity (Schill, 2014; Zerrahn et al., 2018).

Unintended storage cycling distorts these optimal curtailment and storage use patterns, as illustrated in Figure 6B, which shows the case with proportionate SLCR. The RLDC of a model specification with zero SLCR is similarly affected and omitted for the sake of clarity. A larger part of VRE surplus generation is taken up by storage and not curtailed (compare area in F in Figure 6B to area A in Figure 6A). Unintended storage cycling results in energy being charged (area G in Figure 6B) and discharged again in the same period (area H). In contrast to the case without unintended storage cycling, there are no hours left in which VRE surplus is curtailed without storage being charged, which would result in a plateau of the residual load at the zero line. Instead, there are hours in which maximum possible unintended storage losses are generated (plateau I). Here, both storage charging and discharging are at maximum capacity, while the remaining VRE surplus is curtailed. Note that same-period storage cycling is limited not only by the installed storage charging capacity but also by the discharging capacity. In our parameterization, optimal storage discharging capacity is lower than the optimal charging capacity. In hours to the left and right of those in plateau I, unintended storage cycling may also occur. Yet, not the full cycling potential is exploited here. On the right-hand side of plateau I, storage charging capacity is never at the maximum, while storage discharging capacity is never at the maximum on the left-hand side of plateau I. The RLDCs of constraint family (3) models deviate only marginally from the other constraint families.

Optimal dispatch and investment decisions of all technologies are affected by unintended storage cycling, which is most pronounced in settings with zero SLCR. Here, optimal storage charging capacity slightly increases to expand the unintended storage cycling potential (vertical distance between dashed lines in plateau J in Figure 7). Further, the VRE capacity is lower than in models with complete SLCR, yielding an upward shift of the RLDC (area between solid lines in Figure 7). While the shifted VRE surplus energy from the negative part of the RLDC increases, the additionally charged energy is not used to serve demand, but partly removed from the system via unintended storage cycling. As a consequence, conventional generation increases (area K between the dashed lines in Figure 7). This goes along with additional capacity entry of coal in models with zero SLCR, which helps to serve residual load during peak periods (vertical distance between dashed lines in plateau L in Figure 7). Note that, while less pronounced, similar effects also occur in settings with proportionate SLCR. Yet, for better illustration, we contrast RLDCs of models with zero and complete SLCR.

#### Effects on market clearing prices

The merit order ranks available generation technologies in ascending order of variable generation costs. In our cost-minimizing setting, technologies with lowest variable costs are dispatched first to meet the demand. In the absence of electricity storage, the technology that supplies the last unit of demand — the marginal technology — sets the uniform price for the power sector at the intersection of demand and supply. The dual variable $\lambda_{t}$ of the energy balance, Equation (10b), indicates the resulting price, which depends on the optimality condition of the marginal technology. It can be derived from the first-order condition of the underlying optimization model that applies to the generation variable of the marginal technology (Biggar and Hesamzadeh, 2014, Ch.4). The price constitutes the sum of variable costs and the dual variables of constraints that may be binding for the marginal technology.

If storage is deployed, the first-order condition of storage discharging includes variable costs, the dual variable of capacity- and energy-related constraints, and the derivative of the renewable energy constraint (Equations SI.16a and SI.16b). While most of these summands coincide for all model specifications, the derivative of the renewable constraint may vary over both constraint families and SLCR levels (Equations SI.18a–SI.18l).

Similarly, the first-order condition of storage charging determines the price at which storage charges (Equations SI.17a and SI.17b). This is the price that results at the intersection of demand and supply. It may also change across both constraint families and SLCR levels due to a variation in the derivation of the renewable energy constraint with respect to storage charging (Equations SI.19a–SI.19l).

**Figure 8:**
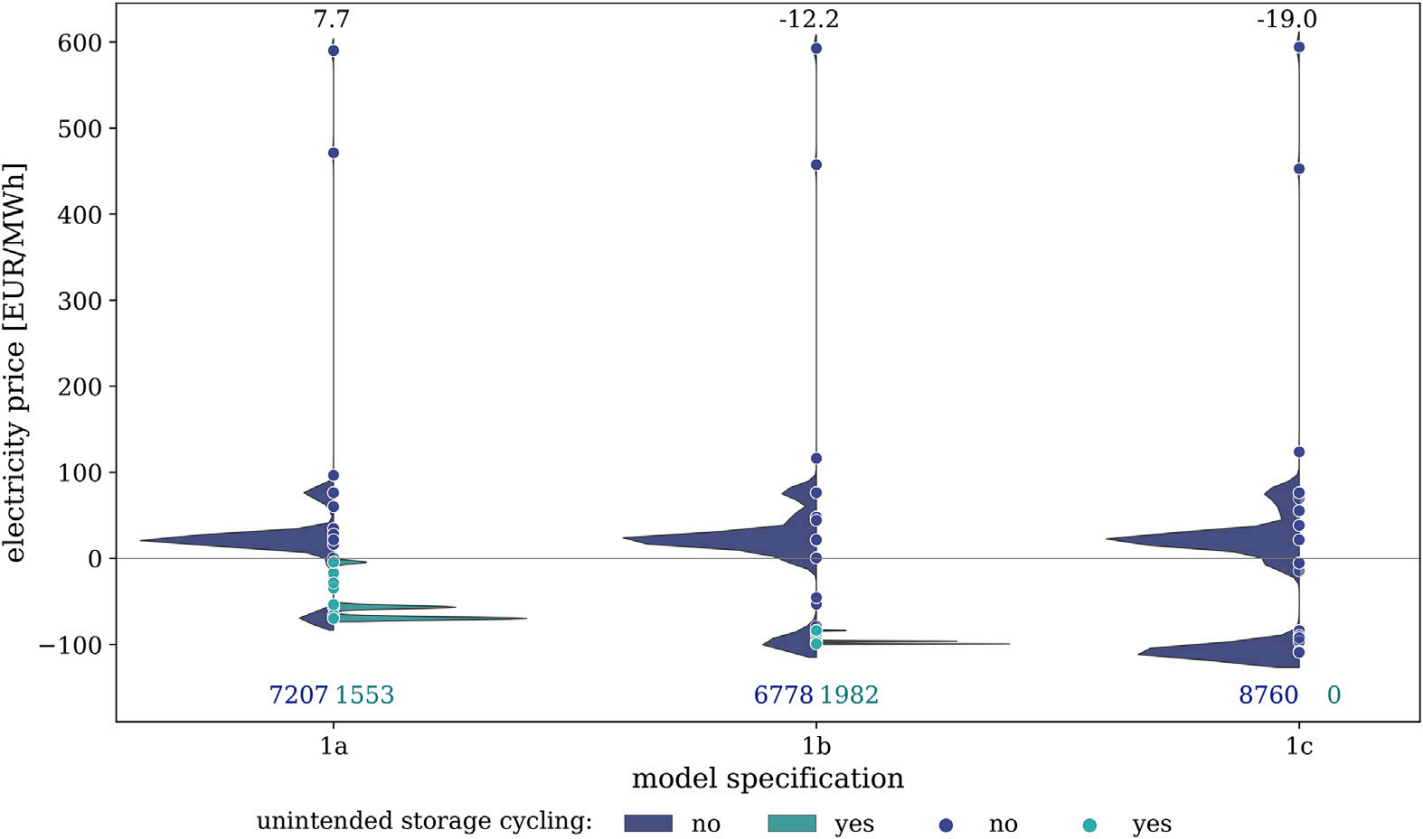
Distribution of prices across different SLCR levels for a minimum renewable share in demand The left side of each violin in dark blue shows the distribution of prices during hours without simultaneous storage charging and discharging. The right side in teal refers to the prices of all hours in which the unintended storage cycling occurs. The bubbles represent underlying discrete prices to visualize accumulations and outliers. The numbers at the top indicate average prices, while the numbers at the bottom refer to the number of hourly prices for each of the violin’s sides.

Figure 8 illustrates the estimated distribution (kernel density estimation) of prices. We interpret the dual variables of the energy balance as hourly wholesale prices, compare Brown and Reichenberg (2021) for model specifications based on constraint family (1). The observed prices vary across SLCR levels for two reasons. First, as the capacity portfolio changes across SLCR levels, the shape of the merit order and the intersection of the supply curve with demand may be affected. Second, varying optimality conditions for storage charging and discharging drive the differences in wholesale prices between different SLCR levels.

Figure 8 also shows that unintended storage cycling occurs only in low-price periods, i.e., in hours with renewable curtailment. Note that prices may become negative in models using constraint family (1), which can be interpreted as the consequence of an energy-based renewable support mechanism (Brown and Reichenberg, 2021; López-Prol and Schill, 2021).

Price formations of models based on constraint families (2) to (4) are characterized by the same variation in the distribution across SLCR levels, but a constant level shift may apply for each constraint family. This effect is due to a variation of optimality conditions of generators across constraint families. It may be explored in more detail in the future, but is beyond the scope of this paper.

#### Drivers of unintended storage cycling

To disentangle the drivers of unintended storage cycling, we conduct sensitivity analyses with respect to key input parameters: the renewable energy target, storage efficiency, as well as the variable costs of storage use, renewable generation, and renewable curtailment.

**Figure 9:**
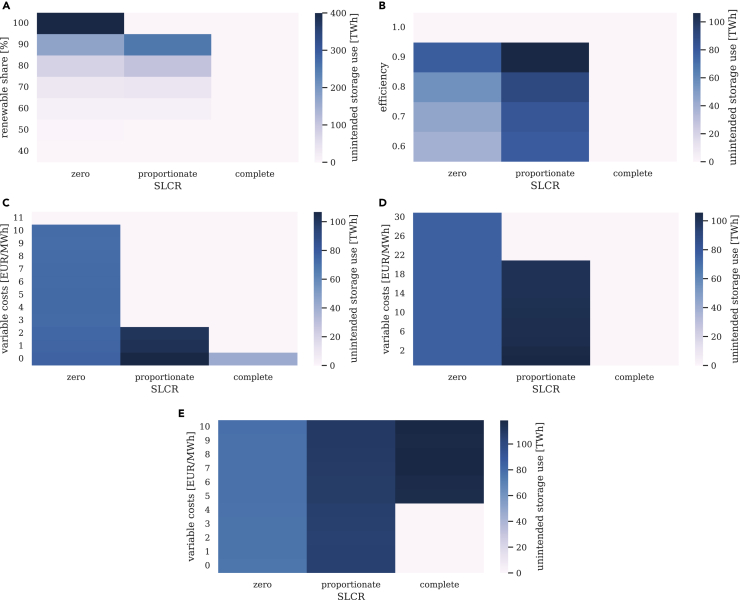
Sensitivity analysis of the annual energy of unintended storage cycling for different SLCR levels A renewable share of 80% applies to all panels except panel 9a. Despite marginal discrepancies, results coincide across all constraint families. (A) Different renewable penetration levels (no occurrence below 40%). (B) Different levels of round-trip storage efficiency. (C) Different levels of variable costs of storage use. (D) Different levels of variable costs of renewable generation (no occurrence above 9000 EUR/MWh for zero SLCR). (E) Different levels of variable costs of renewable curtailment.

**Figure 10:**
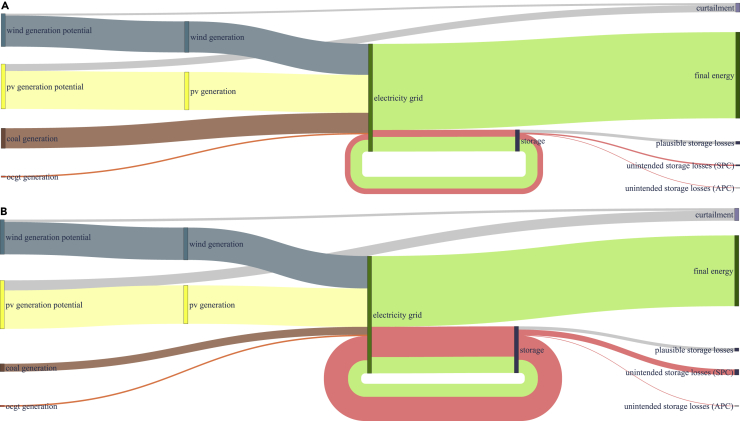
Energy flows for a power sector based on the model (1a) using a renewable share constraint with zero SLCR The height of each trace represents the energy amount. Light green traces represent intended power flows withdrawn from or fed back into the grid. Red traces indicate unintended power flows due to unintended storage cycling. Unintended storage cycling and unintended storage losses grow with the renewable share target. Note that the ratio trace height over represented energy varies across both illustrations due to scaling. (A) Energy flows at a renewable share of 80%. (B) Energy flows at a renewable share of 100%.

With increasing renewable penetration, the number of hours with unintended storage cycling and the energy related to the artifact grow disproportionately (Figure 9, Figure 10A and 10). Below a renewable share of 40%, no storage is deployed due to very limited VRE surplus generation. Accordingly, unintended storage cycling does not occur. As the required VRE penetration increases, VRE surplus energy and optimal electricity storage capacities also grow. Unintended storage cycling becomes available and is increasingly used. In a fully renewable power sector, model specifications with proportionate and complete SLCR converge, because the renewable share constraints coincide (see Table 2 for φ=1). Both of these SLCR levels then prevent unintended storage cycling altogether (see top row in Figure 9A), while model specifications with zero SLCR still suffer from the artifact (see also Figure 10B). By construction, the latter do not comply with the notion of a fully renewable power sector even at a renewable share of 100%, as they do not explicitly require complete storage losses to be covered by renewables. Instead, some level of conventional generation always remains in the system under zero SLCR constraint formulations.

The round-trip efficiency of the electricity storage technology determines storage losses. Yet, unintended storage cycling does not increase with lower round-trip efficiency (below a value of 1.0). Instead, LCOS declines as efficiency increases. The system’s benefit of storage grows, and yields higher optimal storage capacity. This enables higher levels of unintended storage cycling (Figure 9B). A round-trip efficiency of 1.0, a strong (and unrealistic) assumption for electricity storage, averts storage losses and unintended storage cycling altogether.

Increasing variable costs of storage use, attributed to each unit of charging and discharging, are another driver of unintended storage cycling (Figure 9C). If these costs are zero, the economic trade-off between curtailment of VRE surplus and unintended storage losses cannot be quantified. The optimization becomes indifferent between the two options, and unintended storage cycling may occur at undetermined levels. As variable cost for storage use rises, the cost advantage of removing VRE surplus via unintended storage cycling declines. Above a certain threshold, which is substantially higher than the variable costs of current storage technologies (Lacal Arantegui et al., 2014), unintended storage cycling becomes an unfavorable option in the cost minimization. In our stylized setting, this threshold is much lower for models with proportionate SLCR than for models with zero SLCR.

The same holds true for increasing variable costs of VRE generation, which incentivize curtailment of VRE surplus rather than feeding it into the grid. This makes unintended storage cycling less favorable, as converting renewable surplus into additional storage losses increases the variable cost of renewable generation. Above a certain threshold, the costs of unintended storage cycling thus surpass its gains, and the artifact disappears (Figure 9D). Yet, this threshold is beyond of what appears plausible for real-world VRE technologies (Lacal Arantegui et al., 2014).

All results presented so far were generated under the assumption that VRE curtailment comes at no cost. Rising variable costs of curtailment leads to decreasing curtailment levels. Instead, VRE generation at times of high VRE availability increases, which is then removed from the system via additional unintended storage cycling (Figure 9E) in settings with incomplete SLCR. At very high curtailment costs, this becomes effective even for model specifications with complete SLCR.

## Discussion

In this section, we first discuss pros and cons of different possible remedies for unintended storage cycling. We then highlight potential wider implications of the phenomenon in the context of sector coupling and transmission, and finally draw conclusions for modelers.

### A remedy for unintended storage cycling using renewable energy constraints

We have shown that unintended storage cycling can cause significant disruptions of optimal dispatch and investment patterns in models that include a constraint on annual renewable energy generation. To remedy this artifact, the substitution of VRE curtailment with additional storage losses can be prohibited by fully including storage losses to the renewable energy constraint. This is done in all model specifications with complete SLCR, regardless of the constraint family.

Real-world renewable energy targets are often defined as the ratio of annual generation from renewable energy sources over total electricity generation (or total demand) in the system. This implies that storage losses should also be distributed among renewable and conventional generators according to the targeted renewable share. Model specifications with proportionate SLCR conform with this notion, thus appearing as most policy-relevant. In contrast, models with complete SLCR are overly restrictive in the sense that they require additional renewable generation to completely cover storage losses.

Notwithstanding, we recommend applying a renewable energy constraint with complete SLCR, as this remedies unintended storage cycling. In post-optimization, the achieved renewable share could still be reported according to both complete and proportionate SLCR. The latter can then be interpreted and communicated as the actual renewable share of the system, which is slightly higher than the former. For example, in our model setting with complete SLCR and a renewable target of 80%, the actual renewable share reported according to the formulation with proportionate SLCR is 81.16%. We consider such a slightly increased renewable share to be a reasonable price for energy modelers to pay to avoid unintended storage cycling, especially as renewable shares are set to increase anyway in light of ongoing ambitions to achieve carbon neutrality. Moreover, models with proportionate and complete SLCR converge in a fully renewable setting, which, most likely, is required for carbon neutrality.

Alternatively, modelers may attempt to slightly decrease the desired renewable target in models with complete SLCR to achieve a system configuration that meets the desired target reported as per the proportionate SLCR model. For example, we need to specify a renewable share of 79.3% in a model with complete SLCR in order to achieve an actual renewable energy share of 80% reported according to a formulation with proportionate SLCR. However, the identification of the required share as per complete SLCR would require trial-and-error model runs that need to be done for each model parameterization individually. Depending on the scope of the model and its computational burden, this may be infeasible because of significant modeling time and effort, as well as a high number of trails.

Based on the results shown in the section on the drivers of unintended storage cycling, one option to avoid over-restricting energy models with complete SLCR would be to increase the variable costs of storage use and renewable generation. This makes unintended storage cycling less favorable in the sense of the objective function and may allow using a more intuitive constraint formulation with proportionate SLCR. However, this strategy has to be taken with caution. In the real world, variable storage costs are generally low, irrespective of the technology. A manipulation of these costs distorts the relative costs of the available technology portfolio, and thus also optimal dispatch and investment decisions. The deployment of storage may decline, potentially causing far-reaching system-wide effects, such as a hindered integration of VRE. Moreover, identifying an appropriate increase in variable storage costs that does not substantially affect optimal storage deployment while preventing unintended storage cycling is not straightforward. Instead, it requires extensive testing, and may even be infeasible, depending on the model parameterization. We thus advise against the deliberate manipulation of storage cost assumptions to remedy unintended storage cycling when using renewable energy targets.

### Potential remedies for unintended storage cycling using alternative renewable targets

There are two alternative options for avoiding unintended storage cycling while using renewable targets with a basis other than annual energy. First, instead of using the actual renewable energy generation (after curtailment), the theoretical renewable generation potential (before curtailment) could serve as the base element in the renewable energy constraint (see Equation 1) (Gils et al., 2017; Scholz et al., 2017; Child et al., 2019; Bogdanov et al., 2019). Relaxing the renewable energy constraint via unintended storage cycling is then impossible, because the substitution of curtailment with additional storage losses cannot contribute useful energy to the renewable target. However, this approach changes the nature of the renewable energy target. Models constrained this way optimize the system such that there is sufficient VRE capacity to achieve some renewable energy generation *potential* relative to total demand or total generation. Yet, actual renewable generation is not constrained and may be much lower. Modeling scenarios that adhere to binding renewable energy policy targets in terms of actual renewable generation is thus impossible. Furthermore, the composition of the optimal technology portfolio may significantly deviate from models with complete SLCR, as the role of conventional generators could be overestimated, while the need for renewable generation capacity and appropriate balancing options may be underestimated.

Second, an exogenous expansion target for renewable capacity could be used (Hirth, 2013; Pfenninger and Keirstead, 2015; Anke and Möst, 2021). This approach foresees a capacity mix with an exogenous renewable part, while the remaining part is determined endogenously. Because the actual generation of each technology is not optimized to realize a certain share of renewable energy, the latter may be much lower than desired. Owing to this uncertainty of actual renewable generation, binding renewable energy targets cannot be investigated. This could be resolved by a manual determination of exogenous renewable capacity targets to achieve a desired renewable energy target. However, identifying concrete renewable capacities *a priori* is not straightforward and may require a large number of trials. This becomes particularly relevant for comprehensive model settings that include multiple sector coupling technologies, which puts the feasibility of this approach into question.

### Potential remedies for unintended storage cycling using emission constraints

Instead of including renewable targets, modelers may constrain or penalize carbon emissions to achieve high renewable shares and, at the same time, avoid unintended storage cycling. Based on our literature review, we discuss two options: a limitation of the allowable emission budget (carbon cap), and an explicit carbon price.

Given $e_{s}$ being the specific emissions per generator *s* and CB the available carbon budget, a constraint implementing a carbon cap could be formulated as:

(Equation 6) $$\underset{s,t}{\sum }e_{s}G_{s,t}\leq CB$$

The dual variable of this constraint indicates how much total system costs change if the carbon cap tightens by one marginal unit. We interpret this as the system costs for one unit of emission reduction. In a long-term equilibrium, this implicit carbon price can be interpreted as a carbon price paid by emitting generators (Brown and Reichenberg, 2021). Note that setting policy-relevant carbon budgets in electricity models may be challenging, as political targets for the power sector often do not exist.

In contrast, setting an explicit carbon price on each unit of emissions is more easy to implement, and it becomes directly effective as an additional variable cost component in the objective function.

In both settings, unintended storage cycling cannot occur because renewable energy generation is not constrained. The conversion of VRE curtailment into additional storage losses thus never leads to lower-cost solutions. Importantly, this only holds true if variable costs for storage use are greater zero. Otherwise, the model may be indifferent between VRE curtailment and storage losses.

In general, outcomes of models with similar renewable shares may substantially differ, depending on what drives this share, a carbon constraint, or a renewable constraint. The latter promotes additional renewable generation or capacity beyond the unconstrained optimum. This is done by introducing an energy- or capacity-oriented subsidy for renewable generators, which increases their revenues. In contrast, carbon policy instruments impact the use of conventional generators by increasing their costs. This forces a decline especially of those generators with the highest carbon emissions, which may be replaced by lower-emission technologies. The resulting capacity and energy mix may significantly differ from the solution based on a binding renewable target. A binding carbon cap favors lower-emission technologies (e.g., gas-fired plants) over higher-emission technologies (e.g., lignite-fired plants). As the former tend to have higher variable and lower capital costs, this shift may decrease the optimal storage deployment and affect the overall capacity mix.

Explicit carbon pricing affects the merit order of available generation technologies, increasing the variable cost of emitting technologies. Neither renewable energy nor carbon emissions are constrained. The resulting optimal capacity and energy mix resembles the unconstrained optimum that reflects the economics of the available technology portfolio. Depending on the costs and availability of flexibility options in the model, substantial carbon prices may be required to achieve very high shares of renewables. Moreover, the determination of appropriate price levels *a priori* may be difficult because not only economic but also political, environmental, and social aspects may impact price formations on the electricity wholesale market.

While model formulations that constrain or penalize carbon emissions can effectively avoid unintended storage cycling, they generally lead to different outcomes than models with explicit renewable energy targets. Hence, they are not an adequate substitute for cases where the effects of explicit renewable targets are of interest, e.g., in many countries that have set such renewable targets.

### Potential remedy for unintended storage cycling using binary variables

Another potential remedy for unintended storage cycling is to use binary variables that prevent a storage unit to be both charged and discharged at the same time. This could readily be implemented in unit commitment models, which already include binary variables for power plant operation (e.g., PLEXOS, SpineOpt.jl, or Backbone in Table 1). For example, introducing a binary variable $U_{r,t}$ with $U_{r,t}=0$ in times of storage charging and $U_{r,t}=1$ in times of discharging would alter the storage constraint (10g) as follows:

(Equation 7a) $$\begin{array}{ll} G_{r,t}^{in}\leq C_{r}^{in}\left(1-U_{r,t}\right) & \forall r,t \end{array}$$

(Equation 7b) $$\begin{array}{ll} G_{r,t}^{out}\leq C_{r}^{out}U_{r,t} & \forall r,t \end{array}$$

This model formulation is used in Schill et al. (2017a) to avoid same-period storage cycling in a scenario where renewable curtailment is penalized with a cost term in the objective function. In fact, it effectively prevents SPC. Yet, this formulation still allows for APC. For instance, additional storage charging in period *t* combined with additional storage discharging in period t+1 could convert VRE surplus from *t* to unintended storage losses arising in t+1. Although we did not explore this question numerically, we expect that a substantial fraction of SPC could be transformed to APC in such models. Hence, including binary variables may not only provide an incomplete remedy for the artifact but may also make it even more difficult to detect its occurrence and energy volume. At the same time, introducing additional binary variables generally makes solving numerical models more challenging. We leave a further investigation of this aspect to future research.

### Unintended energy losses in settings with sector coupling

The use of renewable electricity for mobility, heating, and industrial processes is considered a main strategy for decarbonizing the energy system (de Coninck et al., 2018). This strategy is often referred to as sector coupling. It also includes indirect electrification via green hydrogen, which may be used where direct electrification is not viable (Jacobson et al., 2017; Hanley et al., 2018).

The mechanism of unintended storage cycling described here for electricity storage may also apply to various sector coupling technologies, as these also come with inherent energy losses. Here, the model artifact may materialize in the form of VRE surplus being converted into unintended energy losses.

There are three relevant types of such losses: First, losses inherent to sector coupling options that allow for re-conversion to electricity, i.e., with an operation principle similar to electricity storage. These take up energy from the power sector and convert it to some intermediate form. The energy is then either consumed for end energy use in some other sector, or, if beneficial from the system perspective, partly fed back into the grid at a later point in time. Examples are battery-electric vehicles with a vehicle-to-grid discharging option, power plants fueled with green hydrogen, or load-shifting activities that incur energy losses. To remedy unintended energy losses, we recommend to adjust the loss term of the renewable energy constraint such that all relevant losses are covered by renewable generators. Only losses related to processes enabling unintended energy cycling are relevant in this context (e.g., losses related to discharging electricity back from an electric vehicle battery to the grid). Losses from energy consumption without re-conversion option (e.g., losses related to actually driving an electric vehicle) are not relevant here, if the respective demand of a sector coupling option is exogenous to the model (see Section SI.8).

Second, there are standing losses through self-discharge. These may occur in various types of energy storage, e.g., in different types of low- and high-temperature heat storage, in liquid hydrogen storage, or in some long-duration electricity storage technologies. The model artifact may reoccur here in the form of distorted storage use patterns that aim at increasing standing losses by stretching the storage period. Again, properly accounting for standing losses in the loss term of the renewable energy constraint would remedy the issue.

Third, there may be unintended losses arising from sub-optimal technology choices. Suppose there are two different sector coupling options available, one of them more energy-efficient than the other one. Optimal use and investment decisions may then skew away from the alternative with lower losses toward the one with higher losses. This would allow more VRE surplus from the power sector to be converted into energy losses that relax the renewable constraint. An example for such alternative technologies is different supply chains of green hydrogen, such as liquid or gaseous hydrogen (Stöckl et al., 2021). We leave it to future research to find a remedy for distortions related to this type of unintended energy losses.

### Unintended energy losses in multi-regional settings with transmission losses

Electricity trade facilitates spatial smoothing of VRE generation and electric load in multi-regional settings, especially in the absence of other flexibility options (Brown et al., 2018b). This causes losses in the transmission grid due to the resistance of power lines. Unintended and excessive shifts of energy across and between nodes or regions may arise to generate additional grid losses that remove VRE surplus from the system instead of curtailing it at its place of origin. The additional VRE generation would then help to relax the renewable energy constraint, which lowers the need for VRE capacity and renders a lower-cost solution possible. However, the exported energy does not or only partly serves demand in the importing region, and generation from other generators needs to serve demand. Unintended transmission losses may thus affect optimal use and investment patterns of generation and transmission infrastructure as well as their spatial allocation across regions.

A model specification with a system-wide renewable share referenced to either total demand or generation accumulated across all regions is particularly prone to unintended transmission losses. This is because the spatial allocation of generation and transmission capacity is not constrained, but freely relocatable across all regions. Optimal VRE generation capacity may increase in regions that have low VRE LCOE due to superior weather conditions. At least, some of the additional renewable surplus could be converted into additional renewable generation here, and, subsequently, removed from the system in form of unintended transmission losses. This decreases the need for VRE capacity installations in regions with high LCOE related to inferior weather conditions. Instead, dispatchable technologies with lower LCOE can serve demand here. This capacity shift across regions may lead to lower-cost solution than settings without unintended transmission losses. To prevent the artifact from occurring, the renewable energy constraint would have to require renewable generators to completely cover all transmission losses arising across all regions.

Unintended transmission losses may also arise in settings with disaggregated renewable targets specific to each region. A capacity and portfolio effect similar to the one caused by unintended storage cycling may arise here, only that VRE surplus is exported and partly converted into unintended transmission losses instead of unintended storage losses. Again, we recommend to add transmission losses to the loss term of the renewable energy constraint. Note that total domestic demand does not include the energy that is lost in the transmission grid. Referenced to domestic demand, the loss term of the renewable energy constraint should thus include total losses associated with imports required to supply domestic demand. In contrast, total domestic generation includes the energy lost in the transmission grid when exporting to other countries. When referencing the domestic renewable energy constraint to some fraction of domestic generation, only a fraction of the transmission losses from exports to other regions are considered. The remainder should be added to the loss term of the renewable energy constraint (cp. the treatment of unintended storage losses in the loss term of constraint (2c) in Table 2). Again, this merits further investigation in future research.

### Limitations of the study

Inherent to the design of the renewable energy share constraints are two overlapping effects. First, effects directly related to unintended storage cycling, and, second, a lower ambition level of the renewable energy constraints of models with zero or proportionate SLCR. Because storage losses can, at least partly, be covered by conventional generators here, these models require lower renewable generation than a specification with complete SLCR by definition. Section SI.7 disentangles the impact of both factors. The factor separation raises complementary insights, but also introduces new complexities. For practical model applications, only the combined effects of varying renewable ambition levels and unintended storage cycling are relevant, which are shown in Figure 5.

### Conclusion

In this paper, we describe and investigate the modeling artifact of unintended storage cycling, which may arise in a wide range of cost-minimizing energy models that make use of binding renewable energy constraints. In such models, it can be beneficial not to curtail renewable surplus energy, but to convert excess electricity into additional storage losses. This artifact can be detected when there are periods with simultaneous storage charging and discharging. The respective increase in renewable generation can be realized without additional renewable capacity installations, thus helping to achieve the renewable energy target at lower costs. This may distort optimal dispatch and investment decisions of all technologies.

We specifically explore the impact of different constraint formulations for renewable energy targets on model outcomes and unintended storage cycling. Using a parsimonious power sector model, we analytically derive intuition for the cause of unintended storage cycling from an energy modeler’s perspective. We further investigate how including storage losses in the renewable energy constraint affects optimality conditions for storage in the long-term equilibrium. Only if storage losses have to be completely covered by renewable generation, unintended storage cycling can be prevented. Loosely parameterizing the model to the German power sector, we show that the artifact can substantially distort optimal dispatch and investment decisions as well as modeled market prices.

To prevent unintended storage cycling, we recommend to completely cover storage losses in the renewable energy constraint, if such a constraint is used in energy models. This comes at the expense of a slight over-restriction of the model concerning the renewable target, but we argue that this is a reasonable price to pay to avoid the artifact. Furthermore, this can be addressed by adjusted reporting and target setting. Alternatively, other types of renewable constraints not based on energy could be used to avoid the problem, but these come with other drawbacks. Likewise, the artifact can be resolved by implementing carbon emission constraints or penalties. Yet, as these change overall power sector outcomes, they may not be suitable for some types of policy-relevant analyses where binding renewable energy targets play a role.

Unintended storage cycling may be considered a special case of a more general phenomenon of unintended energy losses. It may also occur in models with sector coupling technologies and transmission. Here, various types of energy losses could be traded-off against renewable curtailment to relax the renewable energy constraint in least-cost model solutions. In general, including such losses in the renewable constraint may help in many cases. We leave it to future research to explore the feasibility and the limits of this approach for complex models with many sector coupling technologies and transmission.

## STAR★Methods

### Key resources table

**Table:** 

REAGENT or RESOURCE	SOURCE	IDENTIFIER
**Deposited data**		

DIETERpy_reduced	Kittel and Schill (2021)	https://doi.org/10.5281/zenodo.5761969

**Software and algorithms**		

Python 3.6.13	Python Software Foundation	https://www.python.org/
GAMS 35.2.0	GAMS Development Corp	https://www.gams.com/
DIETERpy	Gaete-Morales et al. (2021)	https://doi.org/10.1016/j.softx.2021.100784
DIETERpy_reduced	Kittel and Schill (2021)	https://gitlab.com/diw-evu/dieter_public/dieterpy_reduced

### Resource availability

#### Lead contact

Further information and requests for resources and reagents should be directed to and will be fulfilled by the lead contact, Martin Kittel (mkittel@diw.de).

#### Materials availability

Not applicable.

#### Data and code availability

The input data and model used here are available Zenodo Kittel and Schill (2021) and will be updated and maintained on GitLab: https://gitlab.com/diw-evu/dieter_public/dieterpy_reduced.

### Method details

#### Formal definition of a parsimonious dispatch and investment model

We use a parsimonious power sector optimization model to analytically show how different minimum renewable constraint formulations relate to costs and market values of storage. Adopting a long-run equilibrium perspective, the model minimizes total system costs of a full year. These include annualized investment and annual variable costs of generation and storage technologies. The model covers a single region and abstracts from a spatial resolution and grid congestion. We assume linear cost functions and price-inelastic demand, perfect foresight, and a perfect energy-only market. This set of assumptions renders a long-term market equilibrium with zero profits for market participants. The solution coincides with the optimal allocation determined by a benevolent social planer. The model set-up and derivations are similar to Brown and Reichenberg (2021) and Biggar and Hesamzadeh (2014, Ch.4). Throughout the exposition, capital letters denote endogenous decision variables. Small letters refer to sets and exogenous parameters.

We denote $\underset{t}{\sum }d_{t}$ as the total demand for electricity over all hours *t* of the year. The set of generators consists of firm and variable renewable generators s∈R and firm conventional generators s∈C, while an unspecified *s* applies to the set of all generators. $G_{s\in R,t}$ represents hourly renewable energy generation net of curtailment of renewable surplus $CU_{s,t}$. Equivalently, $G_{s\in C,t}$ is the hourly dispatch of conventional generators. Total annual electricity generation is $\underset{s,t}{\sum }G_{s,t}=\underset{s\in R,t}{\sum }G_{s,t}+\underset{s\in C,t}{\sum }G_{s,t}$. The parameter ${\bar{g}}_{s,t}\in \left[0,1\right]$ renders a time-variant availability of generator *s*. We denote a storage unit *r*, its charging from the grid $G_{r,t}^{in}$, and its discharging back to the grid $G_{r,t}^{out}$. Total storage loss is $\underset{r,t}{\sum }\left(G_{r,t}^{in}-G_{r,t}^{out}\right)$, which has to be covered by either renewable or conventional generators. We denote $i_{s}$ and $i_{r}$ as technology-specific investment costs, $o_{s}$ and $o_{r}$ as operation and maintenance costs accruing for hourly dispatch. $C_{s}$, $C_{r}^{in}$ and $C_{r}^{out}$ denote installed capacity (power rating), and $C_{r}^{l}$ storage energy capacity. The hourly storage energy level is $G_{r,t}^{l}$.

For the sake of readability, we define ∘={l,in,out} and ∗={in,out}, and use the following aggregate cost terms for storage investment and usage in the objective function.

Further information and requests for resources and reagents should be directed to and will be fulfilled by the lead contact, Martin Kittel (mkittel@diw.de).

(Equation 8) $$\underset{r,\circ }{\sum }i_{r}^{\circ }C_{r}^{\circ }=\underset{r}{\sum }i_{r}^{l}C_{r}^{l}+\underset{r}{\sum }i_{r}^{in}C_{r}^{in}+\underset{r}{\sum }i_{r}^{out}C_{r}^{out}$$

(Equation 9) $$\underset{r,t,\text{∗}}{\sum }o_{r}^{\text{∗}}G_{r,t}^{\text{∗}}=\underset{r,t}{\sum }o_{r}^{in}G_{r,t}^{in}+\underset{r,t}{\sum }o_{r}^{out}G_{r,t}^{out}$$

Investment costs (8) comprise annualized infrastructure cost of the storage (dis)charge unit, and storage energy costs. Storage usage cost (9) are variable and accrue whenever a storage unit (dis)charges. We formulate the optimization problem of the stylized power sector as follows:

(Equation 10a) $$\underset{\begin{array}{l} C_{s},G_{s,t},\\ C_{r}^{\circ },G_{r,t}^{\circ } \end{array}}{\text{minimize}}\;\underset{s}{\sum }i_{s}C_{s}+\underset{s,t}{\sum }o_{s}G_{s,t}+\underset{r,\circ }{\sum }i_{r}^{\circ }C_{r}^{\circ }+\underset{r,t,\text{∗}}{\sum }o_{r}^{\text{∗}}G_{r,t}^{\text{∗}}$$

(Equation 10b) $$\text{subject to}\;-d_{t}+\underset{s}{\sum }G_{s,t}+\underset{r}{\sum }G_{r,t}^{out}-\underset{r}{\sum }G_{r,t}^{in}=0⊥\lambda_{t}\;\forall t$$

(Equation 10c) $$G_{s,t}\geq 0⊥{\underline{\mu }}_{s,t}\;\forall s,t$$

(Equation 10d) $${\bar{g}}_{st}C_{s}-G_{s,t}-CU_{s,t}=0⊥\lambda_{s,t}^{CU}\;\forall s\in R,t$$

(Equation 10e) $$C_{s}-G_{s,t}\geq 0⊥{\bar{\mu }}_{s,t}\;\forall s\in C,t$$

(Equation 10f) $$G_{r,t}^{\circ }\geq 0⊥{\underline{\mu }}_{r,t}^{\circ }\;\forall r,t$$

(Equation 10g) $$C_{r}^{\circ }-G_{r,t}^{\circ }\geq 0⊥{\bar{\mu }}_{r,t}^{\circ }\;\forall r,t$$

(Equation 10h) $$G_{r,t}^{l}-G_{r,t-1}^{l}-\eta_{r}^{in}G_{r,t}^{in}+{\left(\eta_{r}^{out}\right)}^{-1}G_{r,t}^{out}=0⊥\lambda_{r,t}^{l}\;\forall r,t$$

(Equation 10i) $$\underset{s\in R,t}{\sum }G_{s,t}-\text{Θ}\geq 0⊥\mu_{\theta }$$

(Equation 10j) $$\text{Ω}-\underset{s\in C,t}{\sum }G_{s,t}\geq 0⊥\mu_{\omega }$$

The objective function (10a) minimizes total fixed and variable costs of all technologies. The market clearing condition (10b) balances supply and demand for electricity at all times. Constraints (10c) and (10f) impose non-negativity. Variable renewable generation is limited by VRE availability, and curtailment of VRE surplus (10days), which comes at no costs. Generation of conventional generators may not exceed installed capacity (10e). Storage (dis)charge and levels may be no larger than installed capacities (10g). Constraint (10h) ensures inter-temporal consistency of storage filling and withdrawal. As storage incurs conversion losses, storage (dis)charge effects on the storage level are corrected by the efficiency rates $\eta_{r}^{in}<1$ and $\eta_{r}^{out}<1$ (10h). To avoid free lunch, we further demand that the storage level in the first and last hour of the year need to be equal $G_{r,0}^{l}=G_{r,T}^{l}$. Constraint (10i) imposes a minimum renewable share, requiring total renewable generation aggregated overall s∈R to achieve at least Θ. In contrast, constraint (10j) imposes a maximum share Ω on total conventional generation aggregated over all s∈C. Constraints (10j) and (10i) are mutually exclusive substitutes. Only one is considered by the optimization problem. Table S1 in the supplemental information provides unequivocal constraint formulations for our 12 model specifications aligned with the notation above. A constraint’s dual variable indicates how much the objective value changes if the constraint relaxed, also called shadow price. $\lambda_{t}$ represents the system’s marginal cost of meeting an additional unit of demand for electricity. $\lambda_{s,t}^{CU}$ represents the change in total cost when marginally changing $CU_{s,t}$. In the optimum, it takes the value of zero. The dual variables ${\underline{\mu }}_{s,t}\geq 0$, ${\bar{\mu }}_{s,t}\geq 0$ and ${\underline{\mu }}_{r,t}^{\circ }\geq 0$, ${\bar{\mu }}_{r,t}^{\circ }\geq 0$, $\lambda_{r,t}^{l}$ are the shadow prices of the capacity and generation constraints of generators and storages, respectively. $\mu_{\theta /\omega }\geq 0$ indicate how much total system cost would increase, if the boundary of the binding constraint was relaxed. That is, if one more (less) unit of electricity from renewables (fossil fuels) had to be supplied. Loosely speaking, it is the marginal cost of tightening the VRE policy.

#### Numerical implementation

In the section on the numerical results for unintended storage cycling, we use a numerical implementation of our power sector model to derive optimal dispatch and capacity expansion decisions. It is a stylized version of the larger Dispatch and Investment Evaluation Tool with Endogenous Renewables (DIETER) capacity expansion model (Zerrahn and Schill, 2017). A similar model set-up has been previously used for analyzing electricity storage needs for renewable energy integration (Zerrahn et al., 2018), for reflections on the changing role of electricity storage in the renewable energy transition (Schill, 2020), and for an illustration of the economics of renewables and electricity storage (López-Prol and Schill, 2021). The implementation used here is integrated in a Python-GAMS interface, which enables Python-based data pre- and post-processing, scenario analysis, and visualization (Gaete-Morales et al., 2021). For transparency and reproducibility, we provide the model code, all input data, and a manual in public repositories under permissive licenses (https://gitlab.com/diw-evu/dieter_development/dieterpy_reduced). We apply the model to a stylized setting loosely parameterized to the German power sector. Annual electricity demand is 520 TWh, which can be supplied by a mix of two renewable technologies (solar PV and wind power) and two conventional generation technologies (hard coal and open-cycle gas turbines). Assumptions on fixed and variable costs can be found in Table S2 in the supplemental information. We further include a generic electricity storage technology that is parameterized to resemble pumped hydro storage with a round-trip efficiency of 80%. The energy- and power-related costs of this technology make its application most plausible for medium-duration storage, which suits the cases studied here well. All individual generation units of a technology are modeled as one technology aggregate. This parsimonious model setup allows to focus on the effects of unintended storage cycling for different levels of renewable penetration. Unless stated otherwise, we use a minimum renewable penetration level of 80%.

## Footnotes

Supplemental information can be found online at https://doi.org/10.1016/j.isci.2022.104002.
