# Supplementary material for: Renewable energy targets and unintended storage cycling: Implications for energy modeling
Source: iScience. 2022 Mar 4;25(4):104002. doi: 10.1016/j.isci.2022.104002 (PMC8991206; doi:10.1016/j.isci.2022.104002)
Supplement: Document S1. Figures S1–S8 and Table S1 [file mmc1.pdf]

iScience, Volume *25*

Supplemental information

Renewable energy targets and unintended storage cycling: Implications for energy modeling

Martin Kittel and Wolf-Peter Schill

SI. Supplementary information

SI.1. Constraint formulations

Table SI.1: Minimum renewable and maximum conventional shares considered in the optimization problem, related to Section 4.3.1.

No.	SLCR	share	constraint
1a	zero	$\Omega = \phi \sum_t d_t$	(10i)
1b	proportionate	$\Omega = \phi\Big(\sum_t d_t + \sum_{r,t}(G^{in}_{r,t} - G^{out}_{r,t})\Big)$	(10i)
1c	complete	$\Omega = \phi \sum_t d_t + \sum_{r,t}(G^{in}_{r,t} - G^{out}_{r,t})$	(10i)
2a	zero	$\Omega = \phi\Big(\sum_{s,t} G_{s,t} - \sum_{r,t}(G^{in}_{r,t} - G^{out}_{r,t})\Big)$	(10i)
2b	proportionate	$\Omega = \phi \sum_{s,t} G_{s,t}$	(10i)
2c	complete	$\Omega = \phi \sum_{s,t} G_{s,t} + (1-\phi)\sum_{r,t}(G^{in}_{r,t} - G^{out}_{r,t})$	(10i)
3a	zero	$\Theta = (1-\phi)\sum_t d_t + \sum_{r,t}(G^{in}_{r,t} - G^{out}_{r,t})$	(10j)
3b	proportionate	$\Theta = (1-\phi)\Big(\sum_t d_t + \sum_{r,t}(G^{in}_{r,t} - G^{out}_{r,t})\Big)$	(10j)
3c	complete	$\Theta = (1-\phi)\sum_t d_t$	(10j)
4a	zero	$\Theta = (1-\phi)\sum_{s,t} G_{s,t} + \phi\sum_{r,t}(G^{in}_{r,t} - G^{out}_{r,t})$	(10j)
4b	proportionate	$\Theta = (1-\phi)\sum_{s,t} G_{s,t}$	(10j)
4c	complete	$\Theta = (1-\phi)\Big(\sum_{s,t} G_{s,t} - \sum_{r,t}(G^{in}_{r,t} - G^{out}_{r,t})\Big)$	(10j)

SI.2. Cost assumptions

SI.3. Mathematical background - Lagrangian function

Suppose that f, g_l, and h_k are affine, continuously differentiable functions. Our constrained optimization problem minimizes the objective function f over decision variables x_i, subject to equality constraints g_l and inequality constraints h_k:

$$\begin{aligned} \underset{x_i}{\text{minimize}} \quad & f(x_i) \\ \text{subject to} \quad & g_l(x_i) = 0 \perp \lambda_l \quad \forall l, \\ & h_k(x_i) \geq 0 \perp \mu_k \quad \forall k \end{aligned} \tag{SI.1}$$

For optimality of a minimization problem with the inequality sign convention as in Equation (SI.1), we require parallel objective and constraint gradients at the tangential point (Simon and Blume, 1994, Ch. 18.5):

Table SI.2: Overview of assumptions on investment, annual fixed and variable costs, related to Section 4.3.2 in STAR⋆Methods.

technology	overnight investment costs [EUR/kW(h)]	annual fixed costs [EUR/kW]	variable costs [EUR/MWh]
coal	1300	25	21.55
ocgt	400	1.5	76.34
pv	390	10.6	-
wind	1000	20	-
storage charging	1.1	-	0.5
storage discharging	1.1	-	0.5
storage energy	80	-	-

$$\nabla f(x_i) = \sum_l \lambda_l \nabla_x g_l(x_i) + \sum_k \mu_k \nabla_x h_k(x_i) \tag{SI.2}$$

Based on the optimality condition and the problem set-up in Equation (SI.1), we establish the Lagrangian function with non-negative multipliers of inequality constraints (Simon and Blume, 1994, Ch. 18.5):

$$\mathcal{L}(x_i, \lambda_l, \mu_k) = f(x_i) - \sum_l \lambda_l g_l(x_i) - \sum_k \mu_k h_k(x_i) \tag{SI.3}$$

With this formulation, μ_k indicates the change in the objective if $h_k(x_i)$ is relaxed. For instance, suppose $h_k(x_i)$ includes a constant c, such that $h_k(x_i) \leq c$. If c marginally increased, that is $h_k(x_i) \leq c+\epsilon$, μ_k represents the corresponding rise in $f(x_i)$. In the optimum, which is characterized by the vector $(x_i^*, \lambda_l^*, \mu_k^*)$, the following four KKT conditions are satisfied. First, stationarity, i.e., there is no feasible direction to improve the objective:

$$\nabla_x \mathcal{L}(x_i^*, \lambda_l^*, \mu_k^*) = \nabla_x f(x_i^*) - \sum_l \lambda_l^* \nabla_x g_l(x_i^*) - \sum_k \mu_k^* \nabla_x h_k(x_i^*) = 0 \tag{SI.4}$$

Second, primal feasibility ensures feasibility of the (in-)equality constraints:

$$g_l(x_i^*) = 0 \tag{SI.5a}$$

$$h_k(x_i^*) \leq 0 \tag{SI.5b}$$

Third, while the multipliers (also called dual variables) of the equality constraints are free, dual feasibility establishes non-negative multipliers for inequality constraints:

$$\mu_k^* \geq 0 \tag{SI.6}$$

Finally, complementary slackness conditions require a positive multiplier $\mu_k^* > 0$ if an inequality constraint is binding, i.e., $h_k(x_i^*) = 0$, or $\mu_k^* = 0$ if the constraint is not binding, i.e., $h_k(x_i^*) < 0$:

$$\mu_k^* h_k(x_i^*) = 0 \tag{SI.7}$$

SI.4. Derivation of optimality conditions in an unconstrained optimum

In the unconstrained optimum of the model introduced in Section 4.3.1, renewable energy constraints (10j) and (10i) do not apply. In this case, KKT stationarity yields the following first-order conditions for storage:

$$\frac{\partial \mathcal{L}}{\partial C_r^{\circ}} = 0 \Rightarrow i_r^{\circ} - \sum_t \bar{\mu}_{s,t}^{\circ} = 0 \tag{SI.8}$$

$$\frac{\partial \mathcal{L}}{\partial G_{r,t}^{out}} = 0 \Rightarrow o_r^{out} - \lambda_t - \underline{\mu}_{r,t}^{out} + \bar{\mu}_{r,t}^{out} - (\eta_r^{out})^{-1} \lambda_{r,t}^{l} = 0 \tag{SI.9}$$

$$\frac{\partial \mathcal{L}}{\partial G_{r,t}^{in}} = 0 \Rightarrow o_r^{in} + \lambda_t - \underline{\mu}_{r,t}^{in} + \bar{\mu}_{r,t}^{in} + \eta_r^{in} \lambda_{r,t}^{l} = 0 \tag{SI.10}$$

$$\frac{\partial \mathcal{L}}{\partial G_{r,t}^{l}} = 0 \Rightarrow -\underline{\mu}_{r,t}^{l} + \bar{\mu}_{r,t}^{l} - \lambda_{r,t}^{l} + \lambda_{r,t+1}^{l} = 0 \tag{SI.11}$$

KKT complementary slackness requires the following for the storage inequality constraints:

$$\underline{\mu}_{r,t}^{\circ} G_{r,t}^{\circ} = 0 \tag{SI.12}$$

$$\underline{\mu}_{r,t}^{\circ} (C_r^{\circ} - G_{r,t}^{\circ}) = 0 \tag{SI.13}$$

We establish the zero-profit rule for storage of the unconstrained optimum as follows:

$$
\begin{aligned}
&\sum_{\circ} i_r^{\circ} C_r^{\circ} + \sum_{t,*} o_r^{*} G_{r,t}^{*} + \sum_t \lambda_t G_{r,t}^{in} \\
&\quad = \sum_{t,\circ} \bar{\mu}_{r,t}^{\circ} C_r^{\circ} + \sum_t G_{r,t}^{out} \Big(\lambda_t + \underline{\mu}_{r,t}^{out} - \bar{\mu}_{r,t}^{out} + \lambda_{r,t}^{l} (\eta_r^{out})^{-1} \Big) \\
&\quad\quad + \sum_t G_{r,t}^{in} \Big(-\lambda_t + \underline{\mu}_{r,t}^{in} - \bar{\mu}_{r,t}^{in} - \lambda_{r,t}^{l} \eta_r^{in} \Big) + \sum_t \lambda_t G_{r,t}^{in} \\
&\quad = \sum_{t,\circ} \bar{\mu}_{r,t}^{\circ} C_r^{\circ} + \sum_t G_{r,t}^{out} \Big(\lambda_t + \underline{\mu}_{r,t}^{out} - \bar{\mu}_{r,t}^{out} + \lambda_{r,t}^{l} (\eta_r^{out})^{-1} \Big) \\
&\quad\quad + \sum_t G_{r,t}^{in} \Big(-\lambda_t + \lambda_t + \underline{\mu}_{r,t}^{in} - \bar{\mu}_{r,t}^{in} - \lambda_{r,t}^{l} \eta_r^{in} \Big) \\
&\quad = \sum_t \Big(\underline{\mu}_{rt}^{out} G_{r,t}^{out} + \underline{\mu}_{rt}^{in} G_{r,t}^{in} + \bar{\mu}_{rt}^{out} (C_r^{out} - G_{r,t}^{out}) + \bar{\mu}_{rt}^{in} (C_r^{in} - G_{r,t}^{in}) \\
&\quad\quad + G_{r,t}^{out} \big(\lambda_t + \lambda_{r,t}^{l} (\eta_r^{out})^{-1} \big) + G_{r,t}^{in} \big(-\lambda_{r,t}^{l} \eta_r^{in} \big) + G_{r,t}^{l} \big(\underline{\mu}_{r,t}^{l} + \lambda_{r,t}^{l} - \lambda_{r,t+1}^{l} \big) \Big) \\
&\quad = \sum_t \Big(\lambda_t G_{r,t}^{out} + \underline{\mu}_{r,t}^{l} G_{r,t}^{l} + \lambda_{r,t}^{l} \big(G_{r,t}^{l} - \eta_r^{l} G_{r,t-1}^{l} - \eta_r^{in} G_{r,t}^{in} + (\eta_r^{out})^{-1} G_{r,t}^{out} \big) \Big) \\
&\quad = \sum_t \lambda_t G_{r,t}^{out}
\end{aligned}
\tag{SI.14}
$$

The first step employs stationarity conditions (SI.8), (SI.9) and (SI.10). The second step rearranges terms, uses complementarity from (SI.13), and substitutes $\underline{\mu}_{r,t}^{l}$ as in (SI.11). Third, the complementarity conditions (SI.12) and (SI.13) are exploited to cancel out terms, while the cycling sum over $G_{r,t}^{l}$ is shifted to relate the timing to $\lambda_{r,t}^{l}$. Last, primal feasibility of (10h) is exploited.

Dividing both sides of Equation (SI.14) by annual energy output of storage $\sum_t G_{s,t}^{out}$ and rearranging terms renders the storage zero-profit condition in the long-term equilibrium:

$$
LCOS_r = MV_r \tag{SI.15}
$$

SI.5. Derivation of optimality conditions with binding renewable targets

When imposing a binding renewable target, the renewable energy constraints (10j) and (10i) become effective. The KKT stationarity conditions (SI.16a) and (SI.16b) refer to models using constraints (10j) or (10i), respectively, and replace the stationarity condition (SI.9) of the unconstrained model. Similarly, (SI.17a) and (SI.17b) replace (SI.10):

$$
\frac{\partial \mathcal{L}}{\partial G_{r,t}^{out}} = 0 \Rightarrow o_r^{out} - \lambda_t - \underline{\mu}_{r,t}^{out} + \bar{\mu}_{r,t}^{out} - (\eta_r^{out})^{-1} \lambda_{r,t}^{l} + \frac{\partial \Theta}{\partial G_{r,t}^{out}} \mu_\theta = 0 \tag{SI.16a}
$$

$$
\frac{\partial \mathcal{L}}{\partial G_{r,t}^{out}} = 0 \Rightarrow o_r^{out} - \lambda_t - \underline{\mu}_{r,t}^{out} + \bar{\mu}_{r,t}^{out} - (\eta_r^{out})^{-1} \lambda_{r,t}^{l} - \frac{\partial \Omega}{\partial G_{r,t}^{out}} \mu_\omega = 0 \tag{SI.16b}
$$

$$\frac{\partial \mathcal{L}}{\partial G_{r,t}^{in}} = 0 \Rightarrow o_r^{in} + \lambda_t - \underline{\mu}_{r,t}^{in} + \bar{\mu}_{r,t}^{in} + \eta_r^{in} \lambda_{r,t}^{l} + \frac{\partial \Theta}{\partial G_{r,t}^{in}} \mu_\theta = 0 \tag{SI.17a}$$

$$\frac{\partial \mathcal{L}}{\partial G_{r,t}^{in}} = 0 \Rightarrow o_r^{in} + \lambda_t - \underline{\mu}_{r,t}^{in} + \bar{\mu}_{r,t}^{in} + \eta_r^{in} \lambda_{r,t}^{l} - \frac{\partial \Omega}{\partial G_{r,t}^{in}} \mu_\omega = 0 \tag{SI.17b}$$

Derivations (SI.16a) and (SI.16b) are alternatives, referring to model configurations based on constraint family (1) and (2) as defined in equation (10i), or constraint family (3) and (4) as in equation (10j), respectively. The same holds true for (SI.17a) and (SI.17b). The optimality conditions for storage discharging are:

$$\Theta_{1a} : \lambda_t = o_r^{out} - \underline{\mu}_{r,t}^{out} + \bar{\mu}_{r,t}^{out} - (\eta_r^{out})^{-1} \lambda_{r,t}^{l} \tag{SI.18a}$$

$$\Theta_{1b} : \lambda_t = o_r^{out} - \underline{\mu}_{r,t}^{out} + \bar{\mu}_{r,t}^{out} - (\eta_r^{out})^{-1} \lambda_{r,t}^{l} - \phi\mu_\theta \tag{SI.18b}$$

$$\Theta_{1c} : \lambda_t = o_r^{out} - \underline{\mu}_{r,t}^{out} + \bar{\mu}_{r,t}^{out} - (\eta_r^{out})^{-1} \lambda_{r,t}^{l} - \mu_\theta \tag{SI.18c}$$

$$\Theta_{2a} : \lambda_t = o_r^{out} - \underline{\mu}_{r,t}^{out} + \bar{\mu}_{r,t}^{out} - (\eta_r^{out})^{-1} \lambda_{r,t}^{l} + \phi\mu_\theta \tag{SI.18d}$$

$$\Theta_{2b} : \lambda_t = o_r^{out} - \underline{\mu}_{r,t}^{out} + \bar{\mu}_{r,t}^{out} - (\eta_r^{out})^{-1} \lambda_{r,t}^{l} \tag{SI.18e}$$

$$\Theta_{2c} : \lambda_t = o_r^{out} - \underline{\mu}_{r,t}^{out} + \bar{\mu}_{r,t}^{out} - (\eta_r^{out})^{-1} \lambda_{r,t}^{l} - (1-\phi)\mu_\theta \tag{SI.18f}$$

$$\Omega_{3a} : \lambda_t = o_r^{out} - \underline{\mu}_{r,t}^{out} + \bar{\mu}_{r,t}^{out} - (\eta_r^{out})^{-1} \lambda_{r,t}^{l} + \mu_\omega \tag{SI.18g}$$

$$\Omega_{3b} : \lambda_t = o_r^{out} - \underline{\mu}_{r,t}^{out} + \bar{\mu}_{r,t}^{out} - (\eta_r^{out})^{-1} \lambda_{r,t}^{l} + (1-\phi)\mu_\omega \tag{SI.18h}$$

$$\Omega_{3c} : \lambda_t = o_r^{out} - \underline{\mu}_{r,t}^{out} + \bar{\mu}_{r,t}^{out} - (\eta_r^{out})^{-1} \lambda_{r,t}^{l} \tag{SI.18i}$$

$$\Omega_{4a} : \lambda_t = o_r^{out} - \underline{\mu}_{r,t}^{out} + \bar{\mu}_{r,t}^{out} - (\eta_r^{out})^{-1} \lambda_{r,t}^{l} + \phi\mu_\omega \tag{SI.18j}$$

$$\Omega_{4b} : \lambda_t = o_r^{out} - \underline{\mu}_{r,t}^{out} + \bar{\mu}_{r,t}^{out} - (\eta_r^{out})^{-1} \lambda_{r,t}^{l} \tag{SI.18k}$$

$$\Omega_{4c} : \lambda_t = o_r^{out} - \underline{\mu}_{r,t}^{out} + \bar{\mu}_{r,t}^{out} - (\eta_r^{out})^{-1} \lambda_{r,t}^{l} - (1-\phi)\mu_\omega \tag{SI.18l}$$

Equivalently, the optimality conditions for storage charging are:

$$\Theta_{1a}: \lambda_t = -o_r^{in} + \underline{\mu}_{r,t}^{in} - \bar{\mu}_{r,t}^{in} - \eta_r^{in}\lambda_{r,t}^{l} \tag{SI.19a}$$

$$\Theta_{1b}: \lambda_t = -o_r^{in} + \underline{\mu}_{r,t}^{in} - \bar{\mu}_{r,t}^{in} - \eta_r^{in}\lambda_{r,t}^{l} - \phi\mu_\theta \tag{SI.19b}$$

$$\Theta_{1c}: \lambda_t = -o_r^{in} + \underline{\mu}_{r,t}^{in} - \bar{\mu}_{r,t}^{in} - \eta_r^{in}\lambda_{r,t}^{l} - \mu_\theta \tag{SI.19c}$$

$$\Theta_{2a}: \lambda_t = -o_r^{in} + \underline{\mu}_{r,t}^{in} - \bar{\mu}_{r,t}^{in} - \eta_r^{in}\lambda_{r,t}^{l} + \phi\mu_\theta \tag{SI.19d}$$

$$\Theta_{2b}: \lambda_t = -o_r^{in} + \underline{\mu}_{r,t}^{in} - \bar{\mu}_{r,t}^{in} - \eta_r^{in}\lambda_{r,t}^{l} \tag{SI.19e}$$

$$\Theta_{2c}: \lambda_t = -o_r^{in} + \underline{\mu}_{r,t}^{in} - \bar{\mu}_{r,t}^{in} - \eta_r^{in}\lambda_{r,t}^{l} - (1-\phi)\mu_\theta \tag{SI.19f}$$

$$\Omega_{3a}: \lambda_t = -o_r^{in} + \underline{\mu}_{r,t}^{in} - \bar{\mu}_{r,t}^{in} - \eta_r^{in}\lambda_{r,t}^{l} + \mu_\omega \tag{SI.19g}$$

$$\Omega_{3b}: \lambda_t = -o_r^{in} + \underline{\mu}_{r,t}^{in} - \bar{\mu}_{r,t}^{in} - \eta_r^{in}\lambda_{r,t}^{l} + (1-\phi)\mu_\omega \tag{SI.19h}$$

$$\Omega_{3c}: \lambda_t = -o_r^{in} + \underline{\mu}_{r,t}^{in} - \bar{\mu}_{r,t}^{in} - \eta_r^{in}\lambda_{r,t}^{l} \tag{SI.19i}$$

$$\Omega_{4a}: \lambda_t = -o_r^{in} + \underline{\mu}_{r,t}^{in} - \bar{\mu}_{r,t}^{in} - \eta_r^{in}\lambda_{r,t}^{l} + \phi\mu_\omega \tag{SI.19j}$$

$$\Omega_{4b}: \lambda_t = -o_r^{in} + \underline{\mu}_{r,t}^{in} - \bar{\mu}_{r,t}^{in} - \eta_r^{in}\lambda_{r,t}^{l} \tag{SI.19k}$$

$$\Omega_{4c}: \lambda_t = -o_r^{in} + \underline{\mu}_{r,t}^{in} - \bar{\mu}_{r,t}^{in} - \eta_r^{in}\lambda_{r,t}^{l} - (1-\phi)\mu_\omega \tag{SI.19l}$$

By way of example, the zero-profit rule for storage in the model specification (1c) is as follows:

$$\begin{aligned}
&\sum_{\circ} i_r^{\circ} C_r^{\circ} + \sum_{t,*} o_r^{*} G_{r,t}^{*} + \sum_{t} \lambda_t G_{r,t}^{in} \\
&= \sum_{t,\circ} \bar{\mu}_{r,t}^{\circ} C_r^{\circ} + \sum_{t} G_{r,t}^{out}\Big(\lambda_t + \underline{\mu}_{r,t}^{out} - \bar{\mu}_{r,t}^{out} + \lambda_{r,t}^{l}(\eta_r^{out})^{-1} - \frac{\partial\Theta_{1c}}{\partial G_{r,t}^{out}}\mu_\theta\Big) \\
&\quad + \sum_{t} G_{r,t}^{in}\Big(-\lambda_t + \underline{\mu}_{r,t}^{in} - \bar{\mu}_{r,t}^{in} - \lambda_{r,t}^{l}\eta_r^{in} - \frac{\partial\Theta_{1c}}{\partial G_{r,t}^{in}}\mu_\theta\Big) + \sum_{t} \lambda_t G_{r,t}^{in} \\
&= \sum_{t,\circ} \bar{\mu}_{r,t}^{\circ} C_r^{\circ} + \sum_{t} G_{r,t}^{out}\Big(\lambda_t + \underline{\mu}_{r,t}^{out} - \bar{\mu}_{r,t}^{out} + \lambda_{r,t}^{l}(\eta_r^{out})^{-1} + \mu_\theta\Big) \\
&\quad + \sum_{t} G_{r,t}^{in}\Big(-\lambda_t + \lambda_t + \underline{\mu}_{r,t}^{in} - \bar{\mu}_{r,t}^{in} - \lambda_{r,t}^{l}\eta_r^{in} - \mu_\theta\Big) \\
&= \sum_{t}\Big(\underline{\mu}_{rt}^{out} G_{r,t}^{out} + \underline{\mu}_{rt}^{in} G_{r,t}^{in} + \bar{\mu}_{rt}^{out}(C_r^{out} - G_{r,t}^{out}) + \bar{\mu}_{rt}^{in}(C_r^{in} - G_{r,t}^{in}) \\
&\quad + G_{r,t}^{out}\big(\lambda_t + \lambda_{r,t}^{l}(\eta_r^{out})^{-1} + \mu_\theta\big) + G_{r,t}^{in}\big(-\lambda_{r,t}^{l}\eta_r^{in} - \mu_\theta\big) \\
&\quad + G_{r,t}^{l}\big(\underline{\mu}_{r,t}^{l} + \lambda_{r,t}^{l} - \lambda_{r,t+1}^{l}\big)\Big) \\
&= \sum_{t}\Big(\lambda_t G_{r,t}^{out} - \mu_\theta\big(G_{r,t}^{in} - G_{r,t}^{out}\big) + \underline{\mu}_{r,t}^{l} G_{r,t}^{l} \\
&\quad + \lambda_{r,t}^{l}\big(G_{r,t}^{l} - \eta_r^{l} G_{r,t-1}^{l} - \eta_r^{in} G_{r,t}^{in} + (\eta_r^{out})^{-1} G_{r,t}^{out}\big)\Big) \\
&= \sum_{t}\Big(\lambda_t G_{r,t}^{out} - \mu_\theta\big(G_{r,t}^{in} - G_{r,t}^{out}\big)\Big)
\end{aligned} \tag{SI.20}$$

The first step employs stationarity conditions (SI.8), (SI.16a) and (SI.17a). Second, the remaining derivatives are computed. The third step rearranges terms, uses complementarity from (SI.13), and

substitutes $\underline{\mu}^l_{r,t}$ as in (SI.11). Fourth, the complementarity conditions (SI.12) and (SI.13) are exploited to cancel out terms, while the cycling sum over $G^l_{r,t}$ is shifted to relate the timing to $\lambda^l_{r,t}$. Last, primal feasibility of (10h) is exploited.

Dividing both sides of equation (SI.20) by the storage unit's annual energy output $\sum_t G^{out}_{s,t}$ and rearranging terms renders its long-term equilibrium condition, as in Table 3:

$$LCOS_r + \mu_\theta NSL_r = MV_r \tag{SI.21}$$

The mathematical proofs zero-profit conditions of all other investigated model specifications follow the same procedure and are available upon request.

SI.6. Specifics of numerical results of constraint family (3)

Dispatch from VRE is more similar between different SLCR levels within constraint family (3) models compared to the other constraint families (Figure SI.1). Furthermore, models based on constraint family (3) slightly favor PV over wind compared to all other models using other constraint families. In contrast, optimal investment decisions coincide across all constraint families, and vary only with respect to the SLCR level (Figure SI.2).

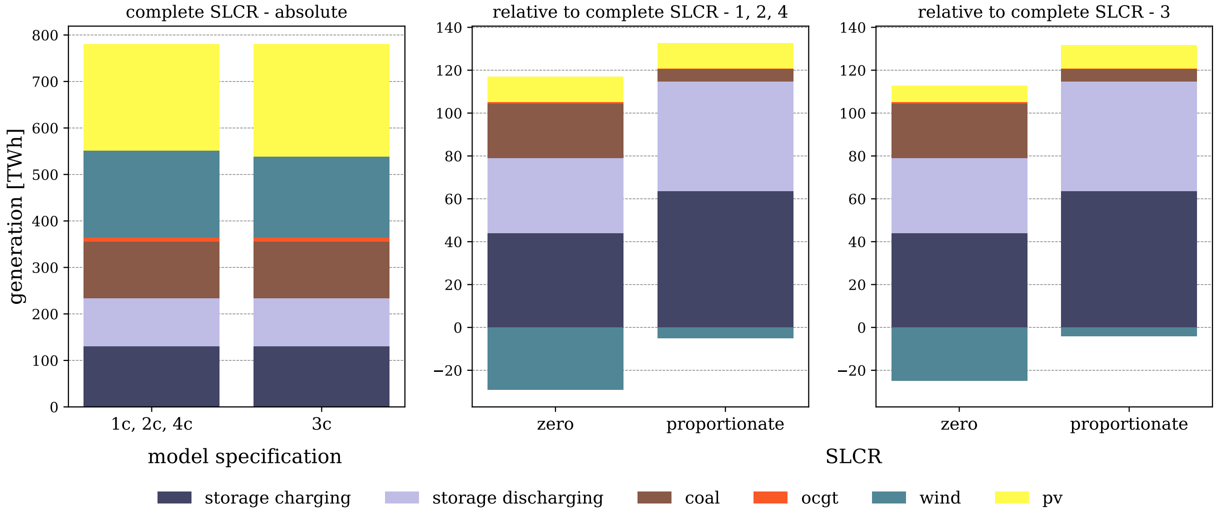

Figure SI.1: Total generation per technology at a renewable target of 80% in absolute numbers for model specifications with complete SLCR (left panel), and the deviation from the complete SLCR case for models with zero and proportionate SLCR for constraint families (1), (2), and (4) (middle panel), and for constraint family (3) (right panel), related to Figure 5.

SI.7. Factor separation: unintended storage cycling and ambition levels of the renewable constraint

The differences in optimal capacity and dispatch decisions presented in Figure 5 are caused by variations of the SLCR level in the renewable energy constraint. Two overlapping factors drive results: (i) unintended storage cycling, and (ii) different ambition levels regarding the required minimum renewable penetration. The latter increases as more storage losses have to be covered by renewable energy. As explained in Section 2.1.1, models with zero SLCR require the lowest use of VRE, followed by models

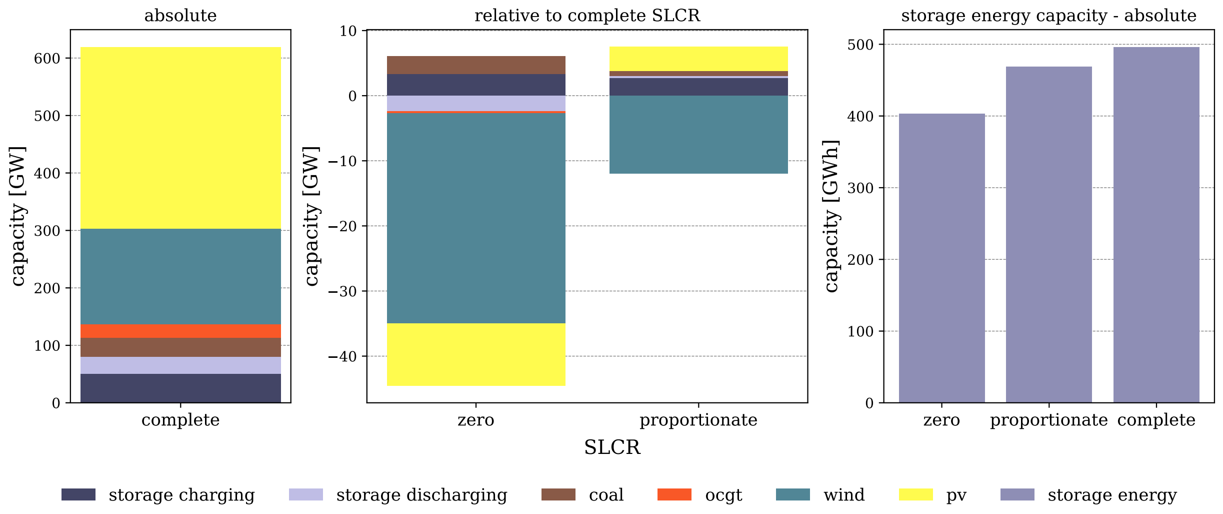

Figure SI.2: Total power capacity per technology at a renewable target of 80% in absolute numbers for model specifications with complete SLCR (left panel), and the deviation from the complete SLCR case for models with zero and proportionate SLCR for all constraint families (middle panel), as well as storage energy capacity in absolute terms for all SLCR levels (right panel), related to Figure 5.

with proportionate SLCR, and such with complete SLCR, which require the highest use of VRE. We disentangle the impacts of both factors by carrying out additional model runs with adjusted renewable energy targets.

First, the effects of unintended storage cycling can be isolated when contrasting a model with incomplete SLCR to a setting with complete SLCR. To rule out the impacts of different ambition levels regarding the renewable target, the realized renewable share of both settings needs to coincide when reported according to the renewable share formula of the model with incomplete SLCR.

In our stylized setting, a complete SLCR model with a 77.0% renewable target achieves the same renewable penetration as a zero SLCR model with an 80.0% renewable target, when reporting both renewable penetration levels according to the zero SLCR specification. Likewise, the renewable penetration of a model with proportionate SLCR and an 80% target matches that of a model with a complete SLCR and a 79.3% target, when reported according to the proportionate SLCR formula. Note that determining such matching renewable targets for complete SLCR settings, in which unintended storage cycling never occurs, may require a time-consuming try-and-error approach, which needs to be carried out for each individual model parameterization.

Second, the impact of different ambition levels of the required renewable energy share can be determined by comparing complete SLCR specifications with different renewable shares. In our stylized setting, contrasting the additional specifications with complete SLCR and a 77.0% or 79.3% renewable target to a complete SLCR model with 80% allows for separating the effects of different ambition levels from unintended storage cycling for zero and proportionate SLCR settings with an 80% renewable penetration, respectively.

Figure SI.3 illustrates the outcomes of the two additional model specifications with adjusted renewable

energy targets (77.0% and 79.3%), which allow for disentangling the effects of both factors. Comparing the cases with complete SLCR, i.e., models without unintended storage cycling, it can be seen that total VRE capacity decreases with lower ambition levels (Figure SI.3a). This decrease is the main driver for lower storage energy capacity needs presented in Section 2.2.2 (Figure SI.3b).

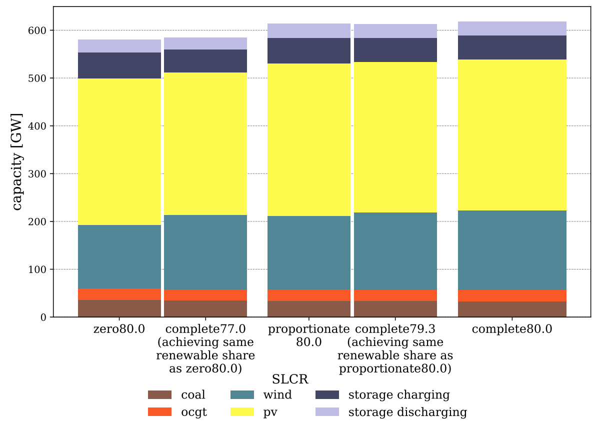

(a) Installed generation capacity including storage charging and discharging.

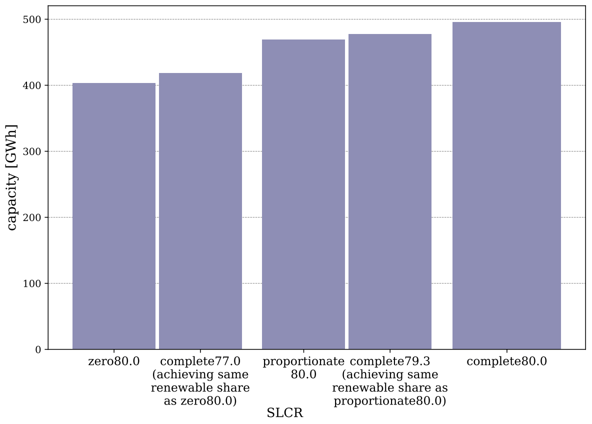

(b) Installed storage energy capacity.

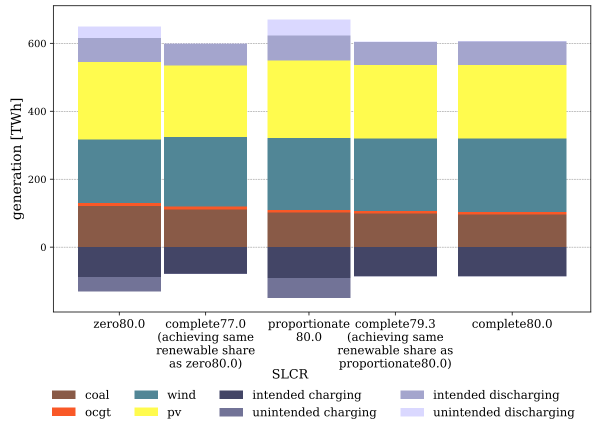

(c) Generation including storage charging (negative part of ordinate) and discharging.

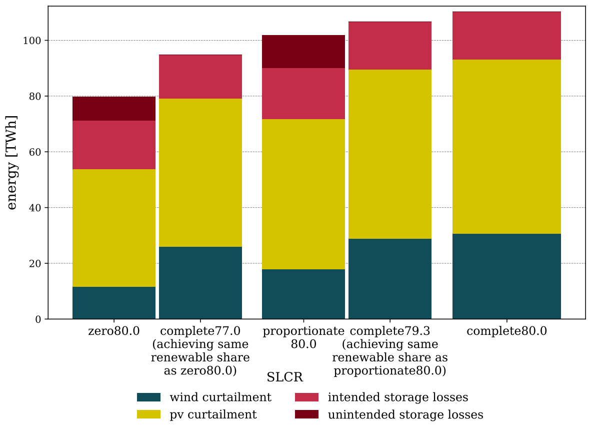

(d) Storage losses and curtailment.

Figure SI.3: Installed capacity, annual generation, curtailment, and storage losses per technology, including factor separation model runs. The numbers in the labels refer to renewable targets specified in respective models. This Figure is related to Figure 5.

Comparing the zero and proportionate SLCR models with their counterparts with complete SLCR and adjusted renewable shares (i.e., comparing columns 1 and 2, as well as columns 3 and 4), it can be seen that unintended storage cycling causes an additional VRE capacity effect, which is somewhat lower than the one attributed to the ambition level (Figure SI.3a). There is also an impact on the optimal VRE portfolio. In total, the need for VRE capacity decreases due to a conversion of curtailment into additional VRE generation that contributes to renewable energy constraint. However, this decrease applies solely to wind, while PV capacity even increases. This is because LCOE of wind power (not accounting for its

generation profile) are higher than those of PV in the model parameterization used here. Generation from VRE is similarly affected (Figure SI.3c). In models with proportionate SLCR, there is a disproportionate shift from wind to PV, and the total VRE generation even increases. The latter is because only a fraction of unintended storage losses contribute to achieving the renewable energy target. Note that the increase in VRE generation between column 4 to column 3 does not incur any costs, as it goes along with lower renewable curtailment. The portfolio effect smooths VRE generation, as PV has a more regular diurnal generation profile than wind power. This contributes to a slightly decreasing need for storage energy capacity (Figure SI.3b).

The need for storage charging and discharging capacity slightly decreases as the renewable ambition level declines (compare columns 2 and 5, as well as columns 4 and 5 in Figure SI.3a). This is most pronounced in models with zero SLCR, and driven by the VRE capacity effect of the lower ambition level. Intended storage use hardly changes with lower ambition levels (Figure SI.3c). In contrast, optimal charging and discharging capacity increases in settings with unintended storage cycling, as it helps to extend the unintended storage cycling potential. Here, additional investment costs for storage capacities are lower than savings due to the VRE capacity and portfolio effect. Intended storage use and storage losses slightly increases with unintended storage cycling driven by these storage capacity effects.

The increase in generation from coal plants in models with incomplete SLCR compared to settings with complete SLCR can be attributed to both the reduced ambition level of the respective renewable constraint and unintended storage cycling, with the former slightly more effective than the latter (Figure SI.3c).

Curtailment reduction is primarily caused by the conversion of curtailment into unintended storage losses (compare columns 1 and 2, as well as 3 and 4 in Figure SI.3d). Another driver is the reduction of optimal VRE capacity caused by a lower ambition level of the renewable target, which leads to lower VRE surplus generation. However, this effect plays less of a role (compare decrease from column 5 to columns 2 and 4 with decrease caused by unintended storage cycling in Figure SI.3d).

SI.8. An example for renewable energy constraints for sector coupling options

In energy models covering the power and transport sectors that include battery-electric vehicles (BEV) with a discharge option (vehicle-to-grid), unintended storage cycling may also arise in vehicle batteries. To prevent this from happening, any possible loss arising from energy cycling not only of stationary electricity storage, but also of BEV (via re-conversion) has to be accounted for in the loss term of the renewable energy constraint. Unavoidable losses associated with charging energy to supply the electrical demand for driving cannot be exploited for additional energy losses, as long as this demand is exogenous to the model. Hence, they can be excluded from loss term. Be v a member of the set of all BEV, $BEV_{v,t}^{in}$ and $BEV_{v,t}^{out}$ charging and discharging from and back into the grid of each BEV in period t, BEV_t^l the battery level, $d_{v,t}^{BEV}$ the required energy for driving, and η_{in}^{BEV} the battery's charging efficiency. Further, to avoid free lunch, we assume that the battery level in the first and last hour of the year need to be equal $BEV_1^l = BEV_T^l$. We recommend to supplement the loss term as displayed in Table 2 with the following concise term:

$$losses^{BEV} = \sum_{v,t} \left(BEV^{in}_{v,t} - BEV^{out}_{v,t} - d^{BEV}_{v,t} / \eta^{BEV}_{in} \right) \tag{SI.22}$$

Losses comprise charged energy reduced by discharged energy and energy for driving, corrected by conversion losses from charging. If the battery level in the first and last period is allowed to diverge, the loss term needs to be corrected by the absolute change in storage level across the entire optimization horizon:

$$losses^{BEV} = \sum_{v,t} \left(BEV^{in}_{v,t} - BEV^{out}_{v,t} - d^{BEV}_{v,t} / \eta^{BEV}_{in} - BEV^{l}_{T} + BEV^{l}_{1} \right) \tag{SI.23}$$

For the loss term (SI.22) without free lunch, the minimal renewable energy constraint (2c) with complete SLCR referenced to total generation, which avoids unintended energy cycling in electricity storage and BEV, would be:

$$\sum_{s \in \mathcal{R},t} G_{s,t} \geq \phi \sum_{s,t} G_{s,t} + (1-\phi) \Big(\sum_{r,t} (G^{in}_{r,t} - G^{out}_{r,t}) + \sum_{v,t} (BEV^{in}_{v,t} - BEV^{out}_{v,t} - d^{BEV}_{v,t} / \eta^{BEV}_{in}) \Big) \tag{SI.24}$$

If the use of multiple vehicle technologies, e.g., BEV, electric vehicle with a hydrogen-fuel fuel cell, or vehicles with an internal combustion engine, is endogenously determined, conversion losses associated with demand for driving need to added to the loss term of the renewable energy constraint, too.
